# BRASSINAZOLE RESISTANT 1 Mediates Brassinosteroid-Induced Calvin Cycle to Promote Photosynthesis in Tomato

**DOI:** 10.3389/fpls.2021.811948

**Published:** 2022-01-20

**Authors:** Xiaowei Yin, Mingjia Tang, Xiaojian Xia, Jingquan Yu

**Affiliations:** ^1^Department of Horticulture, Zhejiang University, Hangzhou, China; ^2^Key Laboratory of Horticultural Plants Growth, Development, and Quality Improvement, Agricultural Ministry of China, Hangzhou, China

**Keywords:** brassinosteroids, BRASSINAZOLE RESISTANT 1 (BZR1), Calvin cycle, Calvin cycle genes, photosynthesis, *Solanum lycopersicum* (tomato)

## Abstract

Calvin cycle is a sequence of enzymatic reactions that assimilate atmospheric CO_2_ in photosynthesis. Multiple components are known to participate in the induction or suppression of the Calvin cycle but the mechanism of its regulation by phytohormones is still unclear. Brassinosteroids (BRs) are steroid phytohormones that promote photosynthesis and crop yields. In this study, we study the role of BRs in regulating Calvin cycle genes to further understand the regulation of the Calvin cycle by phytohormones in tomatoes. BRs and their signal effector BRASSINAZOLE RESISTANT 1 (BZR1) can enhance the Calvin cycle activity and improve the photosynthetic ability. BRs increased the accumulation of dephosphorylated form of BZR1 by 94% and induced an 88–126% increase in the transcription of key genes in Calvin cycle *FBA1, RCA1, FBP5*, and *PGK1*. BZR1 activated the transcription of these Calvin cycle genes by directly binding to their promoters. Moreover, silencing these Calvin cycle genes impaired 24-epibrassinolide (EBR)-induced enhancement of photosynthetic rate, the quantum efficiency of PSII, and *V*_*c,max*_ and *J*_*max*_. Taken together, these results strongly suggest that BRs regulate the Calvin cycle in a BZR1-dependent manner in tomatoes. BRs that mediate coordinated regulation of photosynthetic genes are potential targets for increasing crop yields.

## Introduction

The crop yields need to be increased by 70% to meet the food demand of the world’s population by 2050 (Zhu et al., 2010; Simkin et al., 2019). Enhancing photosynthetic efficiency is one of the crucial approaches to increase crop yields (Long et al., 2015). Photosynthesis is composed of light reactions and dark reactions. The light reactions involve light capture, photochemical reactions, and photosynthetic electron transport chain that drives the formation of ATP and reducing equivalents. The dark reactions mainly involve a series of biochemical reactions in the Calvin cycle, which are affected by temperature, ionic concentration, and light environment. Sugars that produced by photosynthesis can be temporally stored as starch in the chloroplast during the day and consumed at night or be transported to sink tissues to support active growth. When the production of sugars exceeds plants’ demand, the metabolites can feedback downregulate the photosynthetic capacity (Paul and Pellny, 2003). In addition, stomata also influence photosynthesis by gating the entry of CO_2_ into the chloroplast (Lawson and Vialet-Chabrand, 2019; Xiong and Flexas, 2020).

The light reactions and Calvin cycle are two mechanistically different but closely coupled parts. It was reported that the enzymes in the Calvin cycle were activated by reduced thioredoxin (Trx) produced by the photosynthetic electron and proton transport chain (Cheng et al., 2014; Okegawa and Motohashi, 2015). Ruuska et al. (2000) found that decreasing the amount of cytochrome *b/f* complex led to a decrease in the intermediate metabolites in the Calvin cycle (Ruuska et al., 2000). Meanwhile, a missense mutation of the large subunit of Rubisco interrupted the Calvin cycle and suppressed the *de novo* synthesis of D1 and D2 proteins (Takahashi and Murata, 2005). Recently, abundant evidence shows that the regulation of enzyme activity in the Calvin cycle is a key way for crops to improve photosynthetic efficiency (Lin et al., 2014; Simkin et al., 2015, 2017; Driever et al., 2017; Lopez-Calcagno et al., 2020). For example, in tobacco, overexpression of sedoheptulose-1,7-bisphosphatase (SBPase), fructose-1,6-bisphosphate aldolase (FBPase), and cyanobacterial putative inorganic carbon transporter B (ictB) had a positive effect on the photosynthetic rate and biomass accumulation. Besides, altering Rubisco properties by inserting the genes of the Se7942 enzyme and RbcX or CcmM35 can activate Rubisco and increase CO_2_ assimilation in tobacco.

Brassinosteroids (BRs) are a group of steroid hormones that play essential roles in plant growth and development (Nolan et al., 2020). It was known that BRs could induce a phosphorylation cascade and ultimately activate the BR signaling effector, BRASSINAZOLE RESISTANT1 (BZR1) and BRI1 EMS SUPPRESSOR1 (BES1), which are responsible for most of the BR responses (Kim and Russinova, 2020). Activated BZR1 and BES1 can bind to the E-box (CANNTG) and BRRE motif (CGTGT/CG) in the promoters of BR-responsive genes and regulate their expression (He et al., 2005; Yin et al., 2005). Enhancing the endogenous BR biosynthesis specifically in vegetative tissues enlarged the sugar pools in flag leaves and promoted grain filling and yield in rice (Wu et al., 2008). Furthermore, exogenous EBR treatment enhances the plant fitness in response to the suboptimal light and temperature environments (Shu et al., 2016) and nutrient stresses (dos Santos et al., 2020, 2021b). Brassinosteroids can also increase the yields of monocot and dicot crops (Vriet et al., 2012). It is well known that the increase of crop yields is largely attributed to the promotion of photosynthesis. We previously found that BRs enhance photosynthetic CO_2_ assimilation by regulating the ribulose-1,5-bisphosphate (RuBP) carboxylation and regeneration (Yu et al., 2004). Specifically, BRs can activate Rubisco, the rate limiting enzyme of RuBP carboxylation (Spreitzer and Salvucci, 2002). However, whether BRs regulate other enzymes in the Calvin cycle remains unclear and the underlying molecular mechanism needs further studies.

In this study, we show that the BR signaling effector BZR1 plays a crucial role in the regulation of photosynthesis. Enhancement of endogenous BR levels led to increased stability of BZR1, which directly activated the transcription of several Calvin cycle genes including *FBA1, RCA1, FBP5*, and *PGK1* by binding to their promoters. The increase in the transcript abundance of Calvin cycle genes led to the promotion of RuBP carboxylation and regeneration. Meanwhile, silencing of these Calvin cycle genes resulted in the decrease of RuBP carboxylation and regeneration and subsequently compromised BR-induced photosynthesis. In summary, these results provide strong evidence that BRs enhance photosynthesis through BZR1-mediated transcriptional regulation of the Calvin cycle in tomato plants.

## Materials and Methods

### Plant Materials

The tomato (*Solanum lycopersicum* L.) genotype ‘‘Condine Red’’ was used as the wild type (WT) in this study. Its BR synthesis impaired point mutant *dwf* (accession number LA0571), which has a mutation in the BR biosynthetic gene *DWARF*, was obtained from the Tomato Genetics Resource Center (University of California, Davis, CA, United States).^1^ Its BR synthesis increased transgenic plant *DWF*: OE was obtained as described before (Li et al., 2016a). 35Spro:BZR1-HA (transgenic line with HA tag) overexpression line and CRISPR/Cas9 transgenic lines of *bzr1* generated in our laboratory were used in this study (Yin et al., 2018; Wang et al., 2019).

The virus-induced gene silencing (VIGS) constructs for silencing of *FBA1, RCA1, FBP5*, and *PGK1* genes were generated by PCR amplification using specific primers (Supplementary Table 1), digested by *Eco*RI and *Kpn*I, and ligated into empty TRV2 vector who was digested by *Eco*RI and *Kpn*I, too. The recombined plasmids were transformed into *A. tumefaciens* strain GV3101 through electroporation. *A. tumefaciens*-mediated infection was executed as described before (Ekengren et al., 2003). Plants infected by empty TRV2 vector were used as controls in this study, named TRV. All the infected plants were kept at 23°C for 5 weeks before use. Leaflets whose transcript levels were less than 30% of the TRV plants were used in this study (Supplementary Figure 1).

### Growth Conditions and Treatments

Tomato seeds were shaken in a flask at 28°C for 2 days and then sowed into a tray that contains a mixture of peat and vermiculite (2:1/vol:vol). Seedlings were watered with Hoagland nutrition solution every 2 days. When the first true leaf was fully expanded, seedlings were transplanted into plastic pots containing the same matrix and watered with Hoagland nutrition solution every day. The growth conditions in the growth chambers were as follows: a 12-h photoperiod, the temperature of 23°C/20°C (day/night), and PPFD of 300 μmol/m^2^/s photosynthetic photon flux density. Five-week-old seedlings were used for subsequent experiments. The photographs of the phenotype were also taken at this time.

To study how exogenous BR treatment effects photosynthesis, seedlings were sprayed with 24-epibrassinolide (EBR, 200 nM, Sigma, United States) at 8:00 a.m. As EBR was dissolved in ethanol beforehand, seedlings sprayed with distilled water containing the same amount of ethanol were used as controls. Leaf samples were collected 6 h after treatment and frozen in liquid nitrogen immediately. As for seedlings that were used for control, samples were collected at the same time as those EBR-treated plants. All the samples taken were later used for protein extraction or analysis of gene expression.

### Leaf Gas Exchange and Chlorophyll Fluorescence Measurements

The fourth leaf (from top to down) of seedlings at the seven-leaf stage was used for all the measurements in this study. Gas exchange measurements were performed by a portable photosynthesis measurement system (LI-6400; LI-COR, Lincoln, NE, United States) with a fluorescent light source. All the measurements were taken between 8:00 a.m. and 8:00 p.m. The air temperature, relative humidity, CO_2_ concentration, and PPFD used for measurements were as follows: 23°C, 85%, 440 ± 20 μmol/mol and 1,000 μmol/m^2^/s photosynthetic photon flux density and kept for all the cases unless otherwise stated. The CO_2_ assimilation vs. intercellular CO_2_ concentration (A/Ci) curves were fitted as described before (von Caemmerer and Farquhar, 1981). The setting of the CO_2_ concentrations of the A/Ci curves was as follows: 400 μmol/mol at first and then decreased gradually to 50 μmol/mol, afterward returned to 400 μmol/mol for stability and then increased stepwise to 1,500 μmol/mol. The maximum ribulose-1,5-bisphosphate carboxylase/oxygenase (Rubisco) carboxylation rate (*V*_*c,max*_) and maximum ribulose-1,5-bisphosphate(RuBP) regeneration rate (*J*_*max*_) were estimated according to the A/Ci curves using the method described before (Dubois et al., 2007).

The quantum efficiency of PSII (Φ_PSII_), photochemical quenching coefficient (qP), and the efficiency of excitation energy captured by open PSII centers (Fv′/Fm′) were measured using LI-6400 according to the manuscripts supplied by LI-COR. The maximum quantum yield of PSII (Fv/Fm) was measured as described before, using the Dual-PAM-100 system (Walz, Germany) (Li et al., 2016b). Before Fv/Fm was measured, seedlings were put in darkness for adaption for 30 min. For seedlings treated with EBR, gas exchange and chlorophyll fluorescence were measured 24 h after treatment, and A/Ci curves were measured 48 h after treatment.

### Total RNA Extraction and Gene Expression Analysis

Total RNA was extracted from the second leaf (from top to down) of a seven-leaf seedling, following the method described previously (Wang et al., 2020). Total RNA (1 μg) was reverse-transcribed into cDNA as described before (Hu et al., 2021).

Quantitative real-time PCR (qRT-PCR) analysis was conducted in a Light Cycler 480 II real-time PCR system (Roche, Basel, Switzerland) using ChamQ Universal SYBR qPCR Master Mix kits (Vazyme Biotech Co., Ltd., Nanjing, China). Each reaction (20 μL) consists of 10 μl SYBR qPCR Master Mix, 8.2 μl ddH_2_O, 1 μl cDNA, 0.4 μl forward and reverse primer. The PCR condition was started with predenaturation at 95°C for 3 min, followed by denaturation at 95°C for 30 s for 40–45 cycles and then annealing at 58°C for 30 s and extension at 72°C for 1 min. The *Actin* and α*-Tubulin 3* genes were used as internal controls. Gene-specific primers used in this study are shown in Supplementary Table 2. Relative gene expression was estimated as described previously (Livak and Schmittgen, 2001).

### Protein Extraction and Western Blotting

For protein extraction, 0.1 g of leaf samples was harvested and ground into powder in liquid nitrogen. Then, the powder was homogenized in 300 μl 2 × loading buffer containing 0.1 M Tris-Base (pH = 6.8), 10% SDS, 20% glycerol, and the appropriate amount of bromophenol blue. In total, 100 mM dithiothreitol was added just before the use. After fully vortexed, the mixture was heated at 95°C for 10 min and then centrifugated at 4°C, 13,000 g for 10 min. Then, the supernatant was transferred into a new 1.5-ml tube and centrifugated at 4°C, 13,000 g for another 5 min. Subsequently, the extracted proteins were separated by 10% SDS-PAGE and transferred to a nitrocellulose membrane. After that, the membrane was blocked overnight at 4°C in TBST buffer (20 mM Tris, pH 7.5, 150 mM NaCl, and 0.1% Tween 20) with 5% BSA (Amresco 0332). After being washed in TBST at room temperature for half an hour, the membrane was incubated in TBST buffer with 1% BSA containing a rabbit anti-BZR1 multiclonal antibody (Labgic Technology Co., Ltd., Hefei, China) for another 12 h. The secondary antibody was goat anti-rabbit horseradish peroxidase (HRP)-linked antibody (Cell Signaling Technology, Danvers, MA, United States). When the incubation finished, the signal on the membrane was observed following the method described before (Chi et al., 2020).

### Yeast One-Hybrid Assays

Yeast one-hybrid (Y1H) screening assay was conducted using Matchmatch Gold Yeast One-Hybrid System (Clontech) as described before (Fang et al., 2019). The promoters of *FBA1, RCA1, FBP5*, and *PGK1* and the full-length coding region of BZR1 was amplified with specific primers (Supplementary Table 3) and ligated into the pAbAi vector and the pGADT7 vector, respectively. Combined pAbAi vectors were linearized and transformed to Y1H Gold yeast strain together with empty AD vector or pGADT7-BZR1. The transformed yeast cells were screened on SD/Leu-media supplemented with 50 ng/ml or 60 ng/ml aureobasidin A (AbA).

### Dual-Luciferase Assay

Dual-luciferase assay was performed according to the previous study (Min et al., 2012). The full-length coding region of BZR1 was amplified and inserted into the pGreen II 0029 62-SK vector whereas the promoters of *FBA1* (1513 bp), *RCA1* (1953 bp), *FBP5* (1978 bp), and *PGK1* (2017 bp) were cloned and fused into pGreen II 0800-LUC vector. Primers used for conducting vectors are supplied in Supplementary Table 4. Both vectors were electroporated into *A. tumefaciens* strain GV3101. Agrobacterium cultures were infiltrated in MES buffer (10 mM MES, 10 mM MgCl_2_, 150 mM acetosyringone, pH 5.6) and adjusted to an OD600 varied from 0.75 to 0.8. Afterward, agrobacterium culture mixtures of transcription factor and genes (10:1/vol:vol) were infiltrated into *Nicotiana benthamiana* leaves through needle-less syringes. Three days after infiltration, tobacco leaf disks were taken and used for the analysis of the enzyme activity of firefly luciferase (LUC) and renilla luciferase (REN). The empty SK vector mixed with the gene promoters was considered as controls, and the whole test system was performed using the dual-LUC reporter assay system (Promega, Madison, WI, United States).

### Chromatin Immunoprecipitation Assay

Chromatin immunoprecipitation (ChIP) assays were performed with an EpiQuik™ Plant ChIP Kit (Epigentek, Farmingdale, NY, United States) as described previously (Fang et al., 2019). Briefly, 1.5 g of leaf samples was collected from 5-week-old *BZR1*: OE and WT plants. After being fixed in 1% formaldehyde, chromatin was isolated and sonicated into pieces from 150 to 1,000 bp. BZR1 protein with pieces of DNA on it was immunoprecipitated by an anti-HA antibody (Pierce; Rockford, IL, United States). The enriched DNA was amplified by qPCR using specific primers (Supplementary Table 5). Enrichment was calculated by the radio of the immunoprecipitated DNA relative to the input. The goat anti-mouse IgG antibody (Millipore, Darmstadt, Germany) was used as the negative control.

### Statistical Analysis

At least three independent biological replicates sampled from different plants were used for each determination. Statistical analysis of the bioassays was performed with SPSS statistical software (version 19.0, SPSS Inc., Chicago, IL, United States). ANOVA was used to test for significance. The means between different treatments or genotypes were evaluated by Tukey’s test at a level of *p* < 0.05.

### Accession Numbers

Sequence data for the genes studied in this work are available in Sol Genomics Network^2^ under the following accession numbers: *Actin* (Solyc11g005330), α*-Tubulin 3* (Solyc08g006890), *FBA1* (Solyc01g110360), *FBA2* (Solyc02g062340), *FBA3* (Solyc02g084440), *FBA4* (Solyc05g008600), *FBA5* (Soly10g054390), *FBA6* (Solyc07g065900), *FBA7* (Solyc09g009260), *FBA8* (Solyc1 0g083570), *FBA9* (Solyc10g054380), *FBA11* (Solyc09g059040), *PGK1* (Solyc07g066610), *PGK2* (Solyc07g066600), *PGK3* (Solyc0 9g008130), *SBP* (Solyc05g052600), *RCA1* (Solyc09g011080), *RCA2* (Solyc10g086580), *RCA3* (Solyc03g117850), *FBP1* (Solyc1 2g056530), *FBP2* (Solyc04g071340), *FBP3* (Solyc09g011810), *FBP4* (Solyc10g086730), *FBP5* (Solyc10g086720), *FBP6* (Solyc01g106010).

## Results

### Brassinosteroids Enhance the Photosynthetic Capacity of Tomato Plants

**FIGURE 1:**
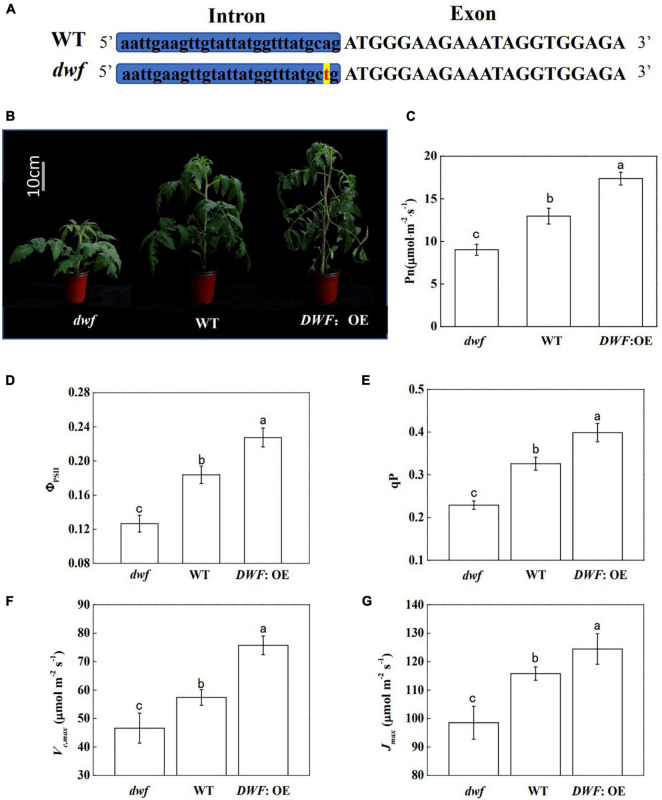
Influence of brassinosteroids (BRs) on plant growth and photosynthesis in tomato plants. BR-deficient mutant (*dwf*), wild type (WT; “Condine Red”), and plants overexpressing the BR biosynthetic *DWARF* gene (*DWF*: OE) were used for this study. Five-week-old plants were used for photographs taken and parameters measured in this analysis. **(A)** The mutation site of *dwf*. **(B)** Plant phenotype. **(C)** Net photosynthetic rate (Pn). **(D)** The actual quantum yield of PSII (Φ_PSII_). **(E)** Photochemical quenching coefficient (qP). **(F)** Maximum ribulose-1,5-bisphosphate carboxylase/oxygenase (Rubisco) carboxylation rate (*V*_*c,max*_). **(G)** Maximum ribulose-1,5-bisphosphate (RuBP) regeneration rate (*J*_*max*_). For **(C–G)**, data are the means ± SD of four biological replicates. Different letters represent significant differences (*p* < 0.05) according to Tukey’s test. Three independent experiments were performed with similar results.

The *dwf* mutant we used in this research is allelic to the strong BR-deficient mutant *d^x^* that is impaired in the BR biosynthesis gene *DWARF* (*DWF*) (Bishop et al., 1999). Additionally, it contains a point mutation in the right border of the 8th intron of *DWF* (from “ag” to “tg”) which affects the mRNA splicing and ultimately leads to protein conformation change (Figure 1A). As indicated by our previous study, the *dwf* mutant contained decreased levels of endogenous BR (Li et al., 2016b). Corresponding to this, the *dwf* mutant showed significant inhibition of plant growth, while overexpression of *DWF* (*DWF*: OE) improved the plant growth (Figure 1B). The growth correlated well with the net photosynthetic rate (Pn) measured at the ambient CO_2_, with the Pn promoted by increased endogenous BR levels (Figure 1C). Similar to Pn, the stomatal conductance (Gs) and transpiration rate (Tr) were significantly decreased in *dwf* plants and were increased in *DWF*: OE plants (Supplementary Figures 2A,C). However, the intercellular CO_2_ concentration (Ci) was not significantly affected by BRs (Supplementary Figure 2B).

Consistent with the Pn, the actual quantum efficiency of PSII (Φ_PSII_) in *dwf* plants was decreased by 31.1%, while the Φ_PSII_ of *DWF*: OE was increased by 23.8% compared to WT (Figure 1D). Photochemical quenching coefficient (qP) showed similar changes to Φ_PSII_ (Figure 1E), whereas the maximum photochemical efficiency of PSII under dark and light (Fv/Fm and Fv′/Fm′), which reflects the intrinsic activity of PSII, was not affected by BR levels (Supplementary Figure 3). Then, we evaluated the photosynthetic capacity of plants with different BR levels by analyzing the curve of CO_2_ assimilation rate vs. Ci (A/Ci). The *in vivo* maximum Rubisco carboxylation rate (*V*_*c,max*_) and RuBP regeneration rate (*J*_*max*_) can be determined by fitting the A/Ci curve according to von Caemmerer and Farquhar’s model (von Caemmerer and Farquhar, 1981). The results showed that defects in BR biosynthesis inhibited *V*_*c,max*_ and *J*_*max*_, whereas overexpression of *DWF* significantly increased *V*_*c,max*_ and *J*_*max*_ compared to WT (Figures 1F,G). Taken together, these results indicated that endogenous BR levels enhance photosynthetic capacity, possibly by regulating the Calvin cycle.

### BRASSINAZOLE RESISTANT 1 Is Involved in the Brassinosteroid-Mediated Enhancement of Photosynthesis

**FIGURE 2:**
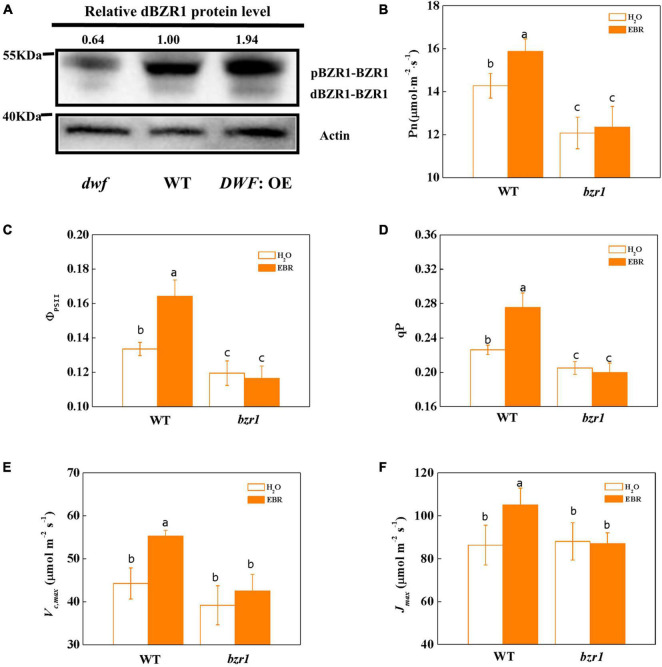
Influence of BZR1 on photosynthesis in tomato plants. **(A)** Induction of dephosphorylation of BZR1 by endogenous BRs. The phosphorylated and dephosphorylated forms of BZR1 are termed by pBZR1 and dBZR1, respectively. Total proteins were probed using an anti-BZR1 multiclonal antibody. The ratio reflects the relative abundance of dBZR1 to total protein, expressed as a number. Actin was used as the loading control. The signal intensity of dBZR1 protein was analyzed with Image Lab. The dBZR1 level of WT plants was set as one. **(B)** Net photosynthetic rate (Pn). **(C)** The actual quantum yield of PSII (Φ_PSII_). **(D)** Photochemical quenching coefficient (qP). **(E)** Maximum ribulose-1,5-bisphosphate carboxylase/oxygenase (Rubisco) carboxylation rate (*V*_*c,max*_). **(F)** Maximum ribulose-1,5-bisphosphate (RuBP) regeneration rate (*J*_*max*_). Five-week-old *bzr1* mutant and wild type (WT; “Condine Red”) plants were used for this analysis. EBR (200 nM) was applied at 8:00 in the morning. For **(B–D)**, values were measured 24 h after EBR treatment. For **(E,F)**, values were measured 48 h after EBR treatment. For **(B–F)**, the data represent the means ± SD of at least three biological replicates. Different letters represent significant differences (*p* < 0.05) according to Tukey’s test. Three independent experiments were performed with similar results.

BRASSINAZOLE RESISTANT 1 (BZR1), as the central transcription factor of the BR signaling pathway, determined the BR signaling output by regulating the expression of hundreds of BR-responsive genes (Wang et al., 2006). In this study, we found that the protein abundance of BZR1, particularly the active dephosphorylated form dBZR1, was decreased in the BR-deficient *dwf* mutant, but was increased by the overexpression of *DWF* (Figure 2A). Similar to the *dwf* mutant, the loss of function *bzr1* mutant showed a decline in photosynthetic capacity, as indicated by lower Pn. Additionally, unlike WT plants, this decline cannot be recovered by 24-epibrassinolide (EBR) application (Figure 2B). Similar to Pn, the Gs and Tr were lower in *bzr1* plants and barely response to EBR treatment (Supplementary Figures 4A,C). However, the intercellular CO_2_ concentration (Ci) was not significantly affected by BZR1 levels or EBR treatment (Supplementary Figure 4B).

BRASSINAZOLE RESISTANT 1 deficiency also resulted in the impaired quantum efficiency of PSII. There was a decline of the Φ_PSII_ and qP in *bzr1* mutant compared to WT plants. Consistent with the enhancement of photosynthesis in *DWF*: OE plants, treatment with EBR promoted the Φ_PSII_ and qP in WT plants, but had no promotion effect on *bzr1* mutant (Figures 2C,D). Consistent with our earlier findings, both Fv/Fm and Fv′/Fm′ were not affected by BZR1 levels or the application of exogenous EBR (Supplementary Figure 5). Besides, *V*_*c,max*_ and *J*_*max*_ in two plant materials showed no significant difference under control conditions but were improved only in WT plants after EBR treatment (Figures 2E,F).

### BRASSINAZOLE RESISTANT 1 Regulates the Transcription of Calvin Cycle Genes

**FIGURE 3:**
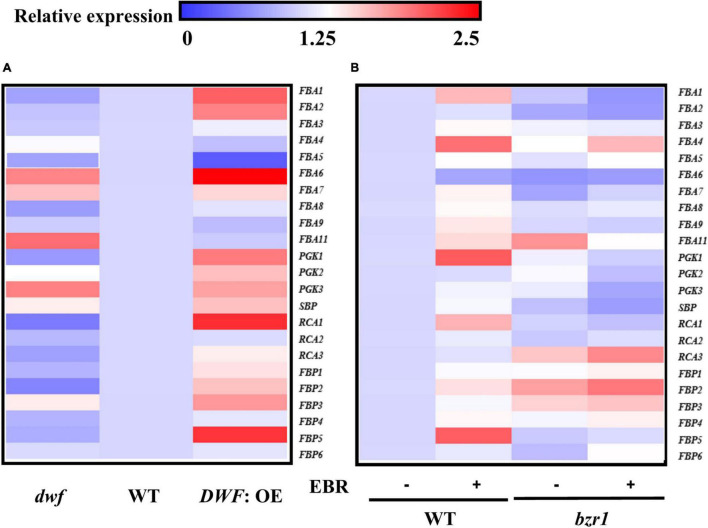
Influence of brassinosteroids (BRs) and BZR1 levels on the transcription of Calvin cycle genes. **(A)** Transcription of Calvin cycle genes in different endogenous BR levels plants. **(B)** Transcription of Calvin cycle genes in different BZR1 levels plants. EBR (200 nM) was applied at 8:00 in the morning. Overall, 6 h after the application, leaf samples were collected for gene expression analysis. *Actin* and α*-tubulin 3* were used as the reference genes here. The data of transcription of genes represent the means ± SD of three biological replicates. Three independent experiments were performed with similar results.

To get a better insight into the mechanism by which BRs regulated photosynthesis, we analyzed the transcription of genes encoding Calvin cycle enzymes, including Rubisco activase (RCA) (3), glycerate-3-phosphate kinase (PGK) (3), fructose-1,6-bisphosphate aldolase (FBA) (10), sedoheptulose-1,7-bisphosphatase (SBPase) (1), and fructose 1,6-bisphosphatase (FBPase) (6). Among these genes, more than half were induced in *DWF*: OE plants, with the accumulation of transcripts increased by 32%-295% as compared to WT plants. Notably, the expression of *FBA1, PGK1, RCA1, RCA3, FBP1, FBP2*, and *FBP5* was consistently suppressed in the *dwf* mutant (Figure 3A). EBR treatment also induced a 24–103% increase in the transcription of *FBA1, FBA3, FBA4, FBA5, FBA7, FBA8, FBA9, FBA11, PGK1, RCA1, FBP2, FBP4*, and *FBP5*, and *FBP5* was most significantly induced by EBR. Among these EBR-induced Calvin cycle genes, *FBA1, FBA7, FBA8, FBA9, PGK1, RCA1*, and *FBP5* could not be transcriptionally induced in *bzr1* mutant (Figure 3B). In combination with those genes regulated by endogenous BR levels, *FBA1, RCA1, FBP5*, and *PGK1* were thought to be regulated by BZR1-mediated BR signaling and were selected for further study.

**FIGURE 4:**
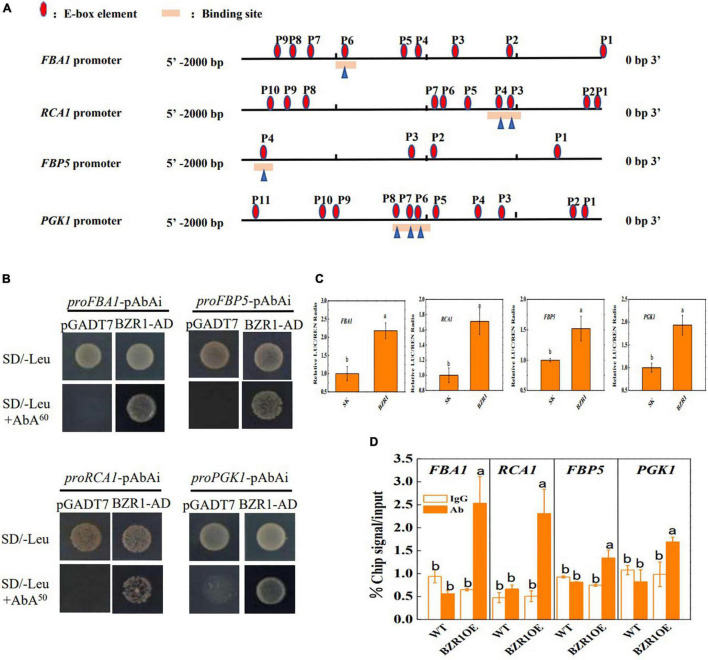
BZR1 binds to the promoters of *FBA1, RCA1, FBP5*, and *PGK1 in vitro* and *in vivo*. **(A)** E-box elements in the promoters of *FBA1, RCA1, FBP5*, and *PGK1* genes. The numbering is counted from the predicted transcriptional start sites. **(B)** Yeast one-hybrid (Y1H) experiment showing the binding of BZR1-AD to the promoters of *FBA1, RCA1, FBP5*, and *PGK1*. Binding sites of every gene were shown in **(A)**. **(C)** Dual-luciferase assay for the regulatory effect of BZR1 on the expression of *FBA1, RCA1, FBP5*, and *PGK1*. The ratio of LUC/REN of the combination of empty SK vectors mixed with the *FBA1, RCA1, FBP5*, and *PGK1* promoters was set as one. **(D)** Direct binding of BZR1 to the promoters of *FBA1, RCA1, FBP5*, and *PGK1* by ChIP–qPCR in 35S-BZR1-HA overexpressing (*BZR1*: OE) plants. Data present the means of three replicates ± SD. Different letters indicate significant differences (*p* < 0.05) according to Tukey’s test. Three independent experiments were performed with similar results.

To further detect the regulation of Calvin cycle genes by BZR1, the promoter regions (∼2 kb) located upstream of the transcriptional start sites of *FBA1, RCA1, FBP5*, and *PGK1* were analyzed and found to contain 4∼11 E-box motifs (CANNTG), the dBZR1 binding sites (Figure 4A). The yeast one-hybrid assay showed that the yeast cells that contained the bait vector combined with the promoter regions of *FBA1, RCA1, FBP5*, and *PGK1* grew on the selective medium when transformed with BZR1-AD; however, when transformed with the empty pGADT7 vector, the yeast cells did not grow on the same selective medium (Figure 4B). This indicates that BZR1 could directly bind to the promoters of *FBA1, RCA1, FBP5*, and *PGK1 in vitro*. The BZR1 binding to these genes was confirmed by dual-luciferase assays. As shown in Figure 4C, the promoter activity of *FBA1, RCA1, FBP5*, and *PGK1* was induced by 1.5- to 2.2-folds by transient overexpression of BZR1. Furthermore, a ChIP-qPCR assay was used to verify the BZR1 binding to the promoters of *FBA1, RCA1, FBP5*, and *PGK1 in vivo*. As shown in Figure 4D, the promoter sequences of *FBA1, RCA1, FBP5*, and *PGK1* were significantly enriched by immunoprecipitation *via* an anti-HA antibody in plants overexpressing the HA-BZR1 recombinant protein but not in WT plants. However, no difference in the efficiency of the pull-down of the promoter sequences was found using the IgG control antibody. Taken together, these results demonstrate that BRs upregulated the transcription of Calvin cycle genes such as *FBA1, RCA1, FBP5*, and *PGK1 via* BZR1 binding to their promoters.

### Silencing of *FBA1, RCA1, FBP5*, and *PGK1* Compromised Brassinosteroid-Induced Photosynthesis

**FIGURE 5:**
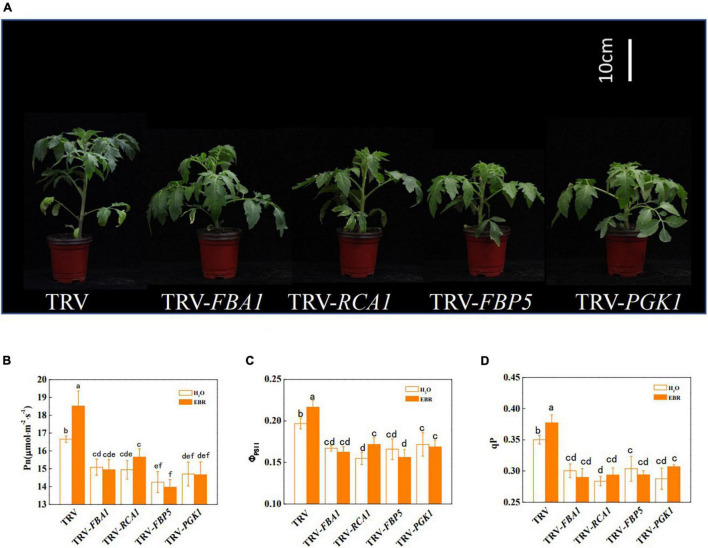
Influence of *FBA1, RCA1, FBP5*, and *PGK1* silencing on plant growth and photosynthesis in tomato plants. **(A)** The phenotype of *FBA1-, RCA1-, FBP5-*, and *PGK1*-silencing plants. **(B)** Net photosynthetic rate (Pn). **(C)** The actual quantum yield of PSII (Φ_PSII_). **(D)** Photochemical quenching coefficient (qP). Five-week-old plants were used for photographs taken and parameters measured in this analysis. Plants, which were infected with an empty TRV2 vector named TRV, were used as controls here. EBR (200 nM) was applied at 8:00 in the morning. For **(B–D)**, values were measured 24 h after EBR treatment. Data are the means ± SD of four biological replicates. Different letters represent significant differences (*p* < 0.05) according to Tukey’s test. Three independent experiments were performed with similar results.

**FIGURE 6:**
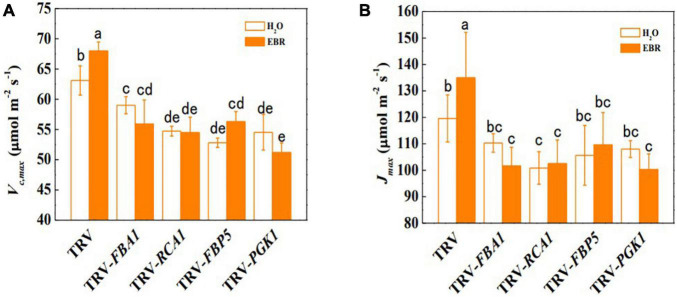
*FBA1, RCA1, FBP5*, and *PGK1* are important in the BR-induced Calvin cycle. **(A)** Maximum ribulose-1,5-bisphosphate carboxylase/oxygenase (RuBisco) carboxylation rate (*V*_*c,max*_). **(B)** Maximum ribulose-1,5-bisphosphate (RuBP) regeneration rate (*J*_*max*_). Five-week-old plants were used in this analysis. EBR (200 nM) was applied at 8:00 in the morning. For **(A)** and **(B)**, values were measured 48 h after EBR treatment. Data are the means ± SD of four biological replicates. Different letters represent significant differences (*p* < 0.05) according to Tukey’s test. Three independent experiments were performed with similar results.

To further clarify whether BRs promote photosynthesis through transcriptional regulation of Calvin cycle genes, the transcription of BZR1-regulated Calvin cycle genes was suppressed through VIGS. The efficiency of gene silencing was confirmed by RT-qPCR and shown in Supplementary Figure 1. The results showed that the suppression of transcription of *FBA1, RCA1, FBP5*, and *PGK1* led to stunted growth and inhibition of photosynthesis (Figure 5 and Supplementary Table 6). Pn, Φ_PSII_, and qP were significantly decreased by gene silencing, whereas Gs, Tr, and Ci were not significantly affected (Figures 5B–D and Supplementary Figure 6). In addition, Fv/Fm and Fv′/Fm′ were not affected by gene silencing, indicating that inhibition of *FBA1, RCA1, FBP5*, and *PGK1* did not result in damages to PSII (Supplementary Figure 7). EBR application enhanced the Pn, Φ_PSII_, and qP in TRV control plants; however, it had no influence on *FBA1-, RCA1-, FBP5*-, or *PGK1-* silencing plants. Interestingly, EBR-induced increase in Gs and Tr was not affected by the silencing of *FBA1*. Meanwhile, silencing of *FBA1, RCA1, FBP5*, and *PGK1* compromised EBR-mediated increase in both *V*_*c,max*_ and *J*_*max*_ (Figure 6A,B). These results indicated that transcriptional regulation of Calvin cycle genes is involved in BR-induced enhancement of photosynthesis.

## Discussion

**FIGURE 7:**
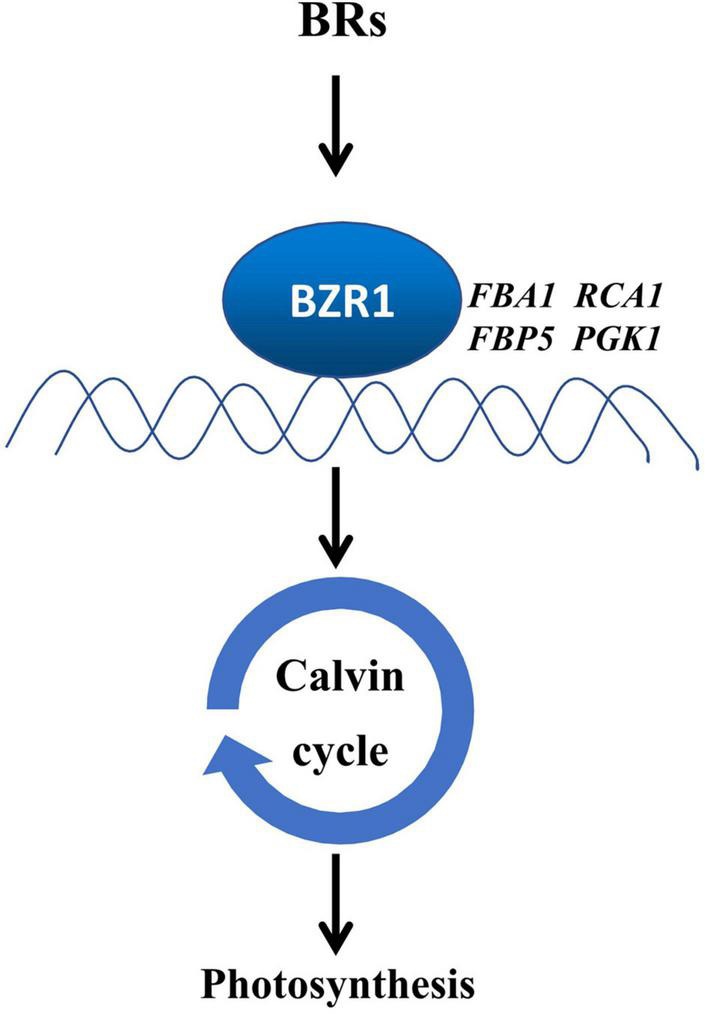
A proposed model for the induction of photosynthesis by BRs in a BZR1-dependent way. BZR1 regulates photosynthesis through the activation of the Calvin cycle in tomatoes. BRs induced the accumulation of BZR1 protein, especially the dephosphorylated forms of BZR1, which activates the transcription of the Calvin cycle genes such as *FBA1, RCA1, FBP5*, and *PGK1* by directly binding to their promoters, subsequently enhancing the Calvin cycle. The induction of the Calvin cycle promotes photosynthesis. Arrows denote positive regulation.

In this study, we provide evidence that BZR1-mediated BR signaling enhanced the photosynthetic capacity of tomato plants by transcriptional regulation of Calvin cycle genes (Figure 7). BRs enhance crop yields by regulating multiple aspects of plant growth and development (Tong and Chu, 2018). Moreover, photosynthesis as the basis of crop yields is also regulated by BRs. The inhibition of BR biosynthesis leads to the decrease in CO_2_ assimilation and the suppression of carbohydrate metabolism (Li et al., 2016b), whereas enhancing the BR biosynthesis increases the sugar pools in leaves and was thought to stimulate the carbon to flow into yield-forming organs (Li et al., 2016a). In this study, we studied the role of BR signaling in the regulation of the Calvin cycle. First, BRs enhanced photosynthetic rate, the quantum efficiency of PSII, and *V*_*c,max*_ and *J*_*max*_ which indicate the state of the Calvin cycle (Figures 1C–G). Second, BRs induced the accumulation of BZR1 (Figure 2A), the central effector of BR signaling, and BZR1 was involved in BR-induced enhancement of photosynthetic capacity (Figures 2B–F). Third, BRs and BZR1 regulated the transcription of Calvin cycle genes such as *FBA1, RCA1, FBP5*, and *PGK1* (Figures 3A,B). Meanwhile, BZR1 can directly bind to the promoters of these Calvin cycle genes and activate their expression (Figure 4). Besides, decreasing the transcription of *FBA1, RCA1, FBP5*, and *PGK1* by VIGS abolished BR-enhanced photosynthesis (Figures 5B–D, 6A,B). All these results demonstrate that BR signaling transcriptionally activates the Calvin cycle in response to photosynthesis.

Our previous studies demonstrated that BRs enhance CO_2_ assimilation by regulating the activity of Rubisco but not by increasing the content of Rubisco (Yu et al., 2004; Xia et al., 2009), and the activation state of Rubisco was associated with the protein level of RCA in plants differing in BR biosynthesis capacity (Li et al., 2016b). RCA is required to release the sugar phosphates from the active site of Rubisco and improve the activity of Rubisco (Portis et al., 2008). The light induction of photosynthesis was accelerated in the transgenic plants with the increased level of RCA and was inhibited by the antisense suppression of the RCA gene (Yamori et al., 2012). These studies demonstrated that regulation of the expression of the *RCA* gene is an effective way of improving photosynthetic efficiency. In this study, we found that BZR1 increased the mRNA level of *RCA1* through direct transcriptional regulation. This can partially explain the BR-induced accumulation of RCA protein (Xia et al., 2009) and suggest that BRs activate Rubisco by regulating the transcription of the *RCA* gene.

Similar to *RCA1*, other Calvin cycle genes such as *FBA1, FBP5*, and *PGK1* were also transcriptionally regulated by BR signaling, which might increase the activities of these enzymes. PGK catalyzes the conversion of the RuBP carboxylation product 3-phosphoglycerate to 1,3-glyceraldehyde diphosphate, which may play a role in the metabolic feedback regulation of Rubisco. FBA and FBPase control metabolic flux in the regeneration of RuBP. Transgenic plants with increased activities of FBA and FBPase showed improved CO_2_ assimilation and biomass yield (Ma et al., 2007). In contrast, VIGS silencing of *FBA1* and *FBP5* in tomatoes inhibited photosynthetic capacity and plant growth (Figures 5A,B). In this and previous studies, BRs have been shown to induce an increase in *J*_*max*_, which reflects the regeneration of RuBP in the Calvin cycle (Yu et al., 2004; Xia et al., 2009; Li et al., 2016b). The results suggested that BR signaling may involve in the control over the carbon flow through the Calvin cycle by regulating the activities of FBA and FBPase. It should be noted that *J*_*max*_ was only slightly affected by single gene silencing of FBA and FBPase. It is likely that FBA and FBPase-mediated reactions are connected and both are required to support the full activation of the Calvin cycle. At ambient growth conditions in this study, the photosynthesis in the silencing plants was still limited as indicated by the reduced *V*_*c,max*_, which ultimately led to a stunning growth. Taken together, BR signaling enhances both *V*_*c,max*_ and *J*_*max*_ through coordinate regulation of key genes controlling multiple sites in the Calvin cycle.

The all-year-round production in the greenhouse is challenged by frequent changes in light and temperature conditions. The photosynthesis of warm-climate crops is sensitive to low temperature and dim light in winter and early spring. The inhibition of CO_2_ assimilation resulted from reduced light utilization efficiency, and low temperature leads to serious impairment of crop yields. The regulation of *RCA* by BZR1 is of particular importance under these conditions, since the increase in RCA protein level enhanced the photosynthetic efficiency at low temperature and weak light (Bi et al., 2017). In addition, BRs and BZR1 positively regulate the photoprotection mechanism (Fang et al., 2019). Besides, the application of EBR can alleviate the damage caused by drought and nutrient stresses (dos Santos et al., 2020, 2021a; Barros Junior et al., 2021; Sousa et al., 2021). Thus, BRs increase the fitness of crops in response to a fluctuating environment, and BR signaling is a promising target of engineering and/or gene editing for sustainable production of greenhouse crops. It should be noted that the enzymes of Calvin cycle are activated *via* redox regulation. Cheng et al. (2014) showed that silencing of chloroplast thioredoxins genes inhibited the activation of Calvin cycle enzymes and impaired the BR-induced increase in CO_2_ assimilation (Cheng et al., 2014). Brassinosteroids have also been shown to interact with the reactive oxygen species (ROS) signaling (Tian et al., 2018). Importantly, BRs induced ROS production *via* BZR1-dependent transcriptional regulation of RESPIRATORY BURST OXIDASE HOMOLOG (RBOH) (Yan et al., 2020), while ROS induced a reducing cellular redox status that increases the stability of RCA (Jiang et al., 2012). Hence, BRs are likely to involve in the posttranslational regulation of Calvin cycle enzymes. Photosynthesis also undergoes feedback regulation by carbon state, which is determined by sugar synthesis, metabolism, and transport (Paul and Foyer, 2001). In addition, carbon and nitrogen metabolism in plants are interconnected. Recently, BR signaling was shown to impinge on the nitrate response pathway (Chu et al., 2021). It is unclear whether nitrogen metabolism is involved in BR-mediated increase in photosynthesis. We propose that BRs regulate photosynthesis through the Calvin cycle, and further studies are required to get a whole picture of how BR signaling regulates photosynthesis and C/N metabolism to fuel plant growth.

## Data Availability Statement

The original contributions presented in the study are included in the article/Supplementary Material, further inquiries can be directed to the corresponding author.

## Author Contributions

JY conceived, designed, and supervised the experiments. XY conducted the experiments and analyzed the data. XY and XX prepared the first draft. MT contributed to the final editing of the manuscript. All authors contributed to the article and approved the submitted version.

## Conflict of Interest

The authors declare that the research was conducted in the absence of any commercial or financial relationships that could be construed as a potential conflict of interest.

## Publisher’s Note

All claims expressed in this article are solely those of the authors and do not necessarily represent those of their affiliated organizations, or those of the publisher, the editors and the reviewers. Any product that may be evaluated in this article, or claim that may be made by its manufacturer, is not guaranteed or endorsed by the publisher.

## Footnotes

1
http://tgrc.ucdavis.edu

2
https://solgenomics.net/
