# Supplementary material for: BRASSINAZOLE RESISTANT 1 Mediates Brassinosteroid-Induced Calvin Cycle to Promote Photosynthesis in Tomato
Source: Front Plant Sci. 2022 Jan 20;12:811948. doi: 10.3389/fpls.2021.811948 (PMC8810641; doi:10.3389/fpls.2021.811948)
Supplement: Supplementary file 1 [file Presentation_1.pptx]

## Slide 1
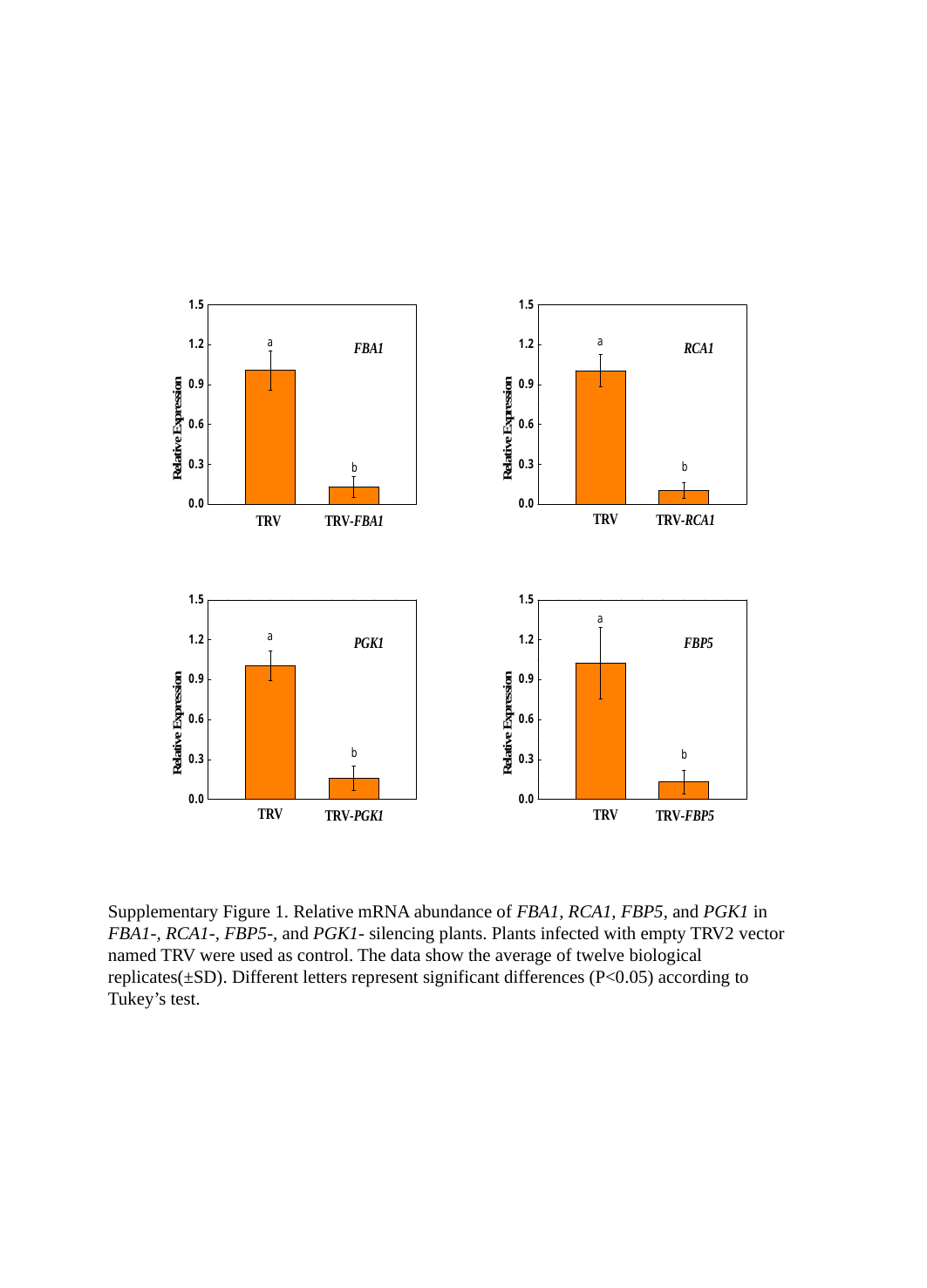

Supplementary Figure 1. Relative mRNA abundance of FBA1, RCA1, FBP5, and PGK1 in FBA1-, RCA1-, FBP5-, and PGK1- silencing plants. Plants infected with empty TRV2 vector named TRV were used as control. The data show the average of twelve biological replicates(±SD). Different letters represent significant differences (P<0.05) according to Tukey’s test.

## Slide 2
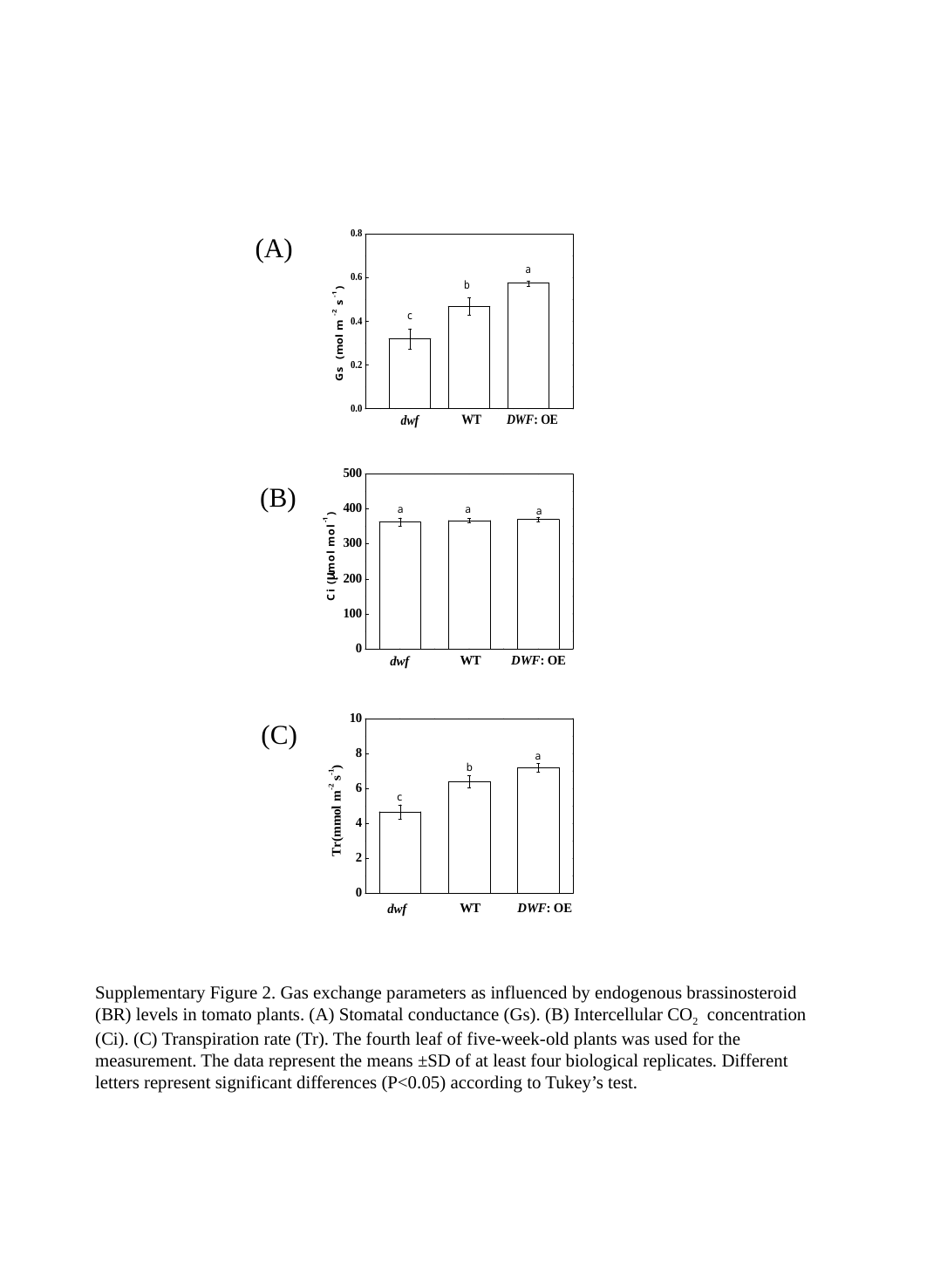

(A)
(B)
(C)
Supplementary Figure 2. Gas exchange parameters as influenced by endogenous brassinosteroid (BR) levels in tomato plants. (A) Stomatal conductance (Gs). (B) Intercellular CO2 concentration (Ci). (C) Transpiration rate (Tr). The fourth leaf of five-week-old plants was used for the measurement. The data represent the means ±SD of at least four biological replicates. Different letters represent significant differences (P<0.05) according to Tukey’s test.

## Slide 3
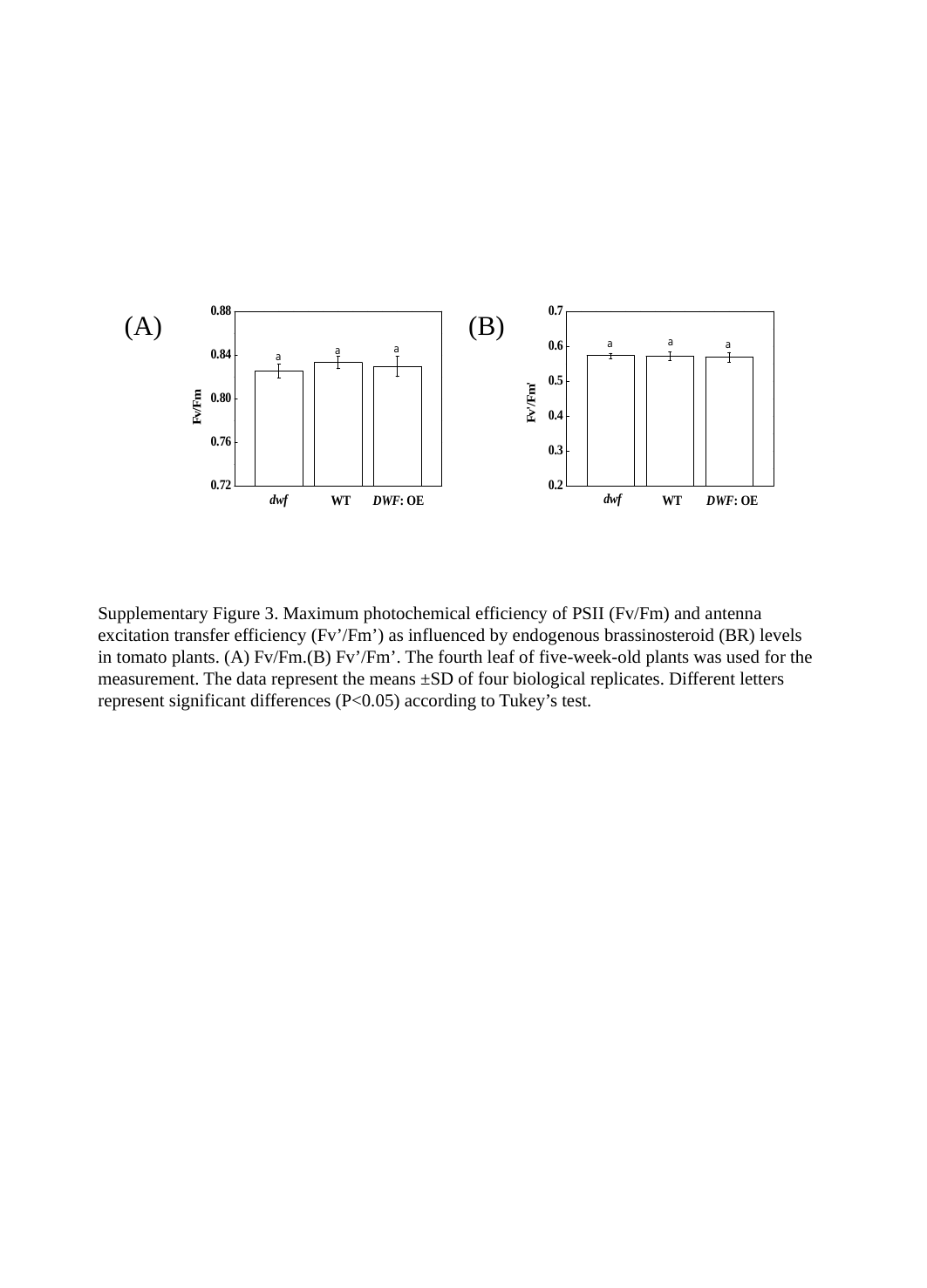

(A)
(B)
Supplementary Figure 3. Maximum photochemical efficiency of PSII (Fv/Fm) and antenna excitation transfer efficiency (Fv’/Fm’) as influenced by endogenous brassinosteroid (BR) levels in tomato plants. (A) Fv/Fm.(B) Fv’/Fm’. The fourth leaf of five-week-old plants was used for the measurement. The data represent the means ±SD of four biological replicates. Different letters represent significant differences (P<0.05) according to Tukey’s test.

## Slide 4
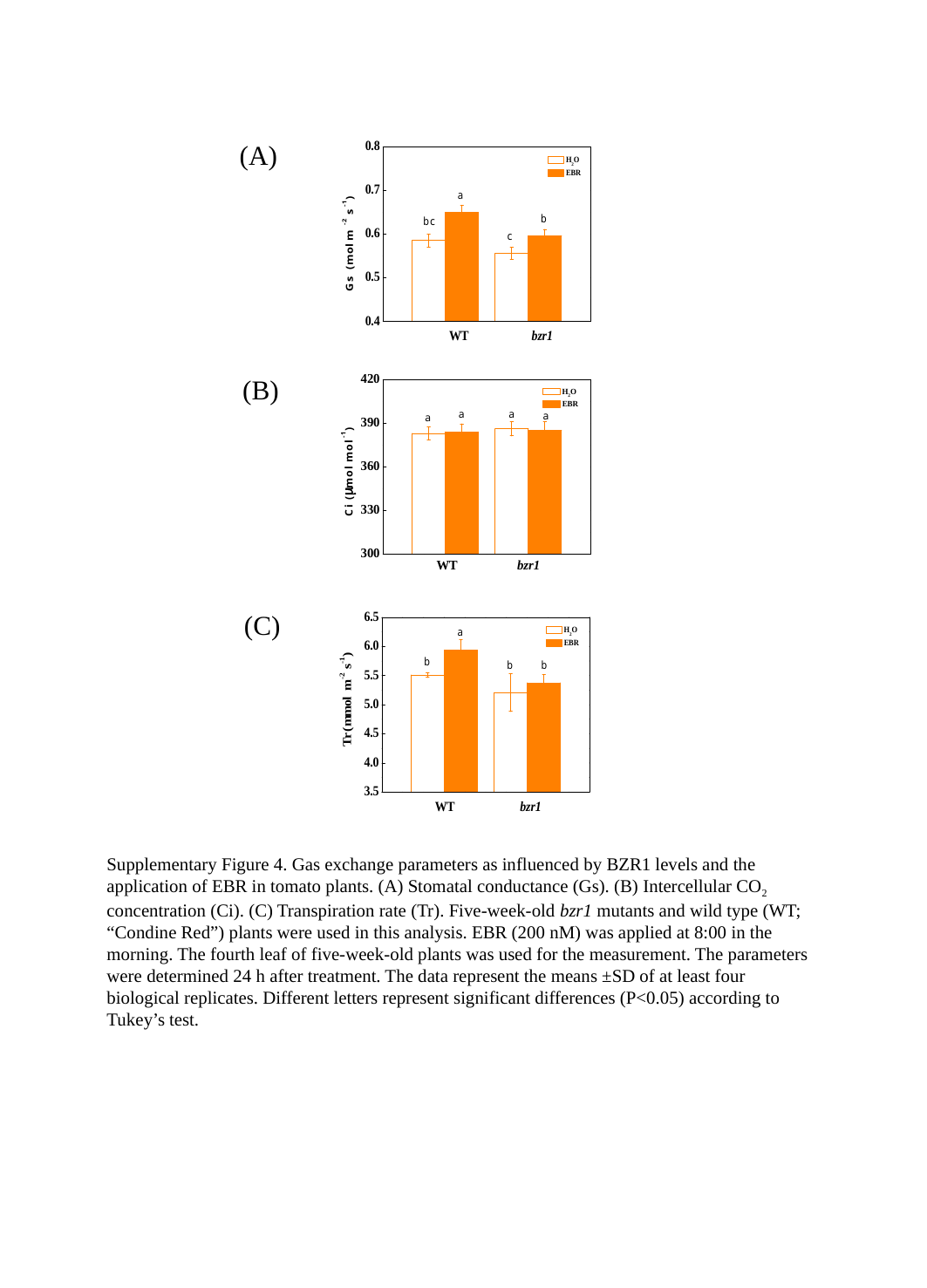

(A)
(B)
(C)
Supplementary Figure 4. Gas exchange parameters as influenced by BZR1 levels and the application of EBR in tomato plants. (A) Stomatal conductance (Gs). (B) Intercellular CO2 concentration (Ci). (C) Transpiration rate (Tr). Five-week-old bzr1 mutants and wild type (WT; “Condine Red”) plants were used in this analysis. EBR (200 nM) was applied at 8:00 in the morning. The fourth leaf of five-week-old plants was used for the measurement. The parameters were determined 24 h after treatment. The data represent the means ±SD of at least four biological replicates. Different letters represent significant differences (P<0.05) according to Tukey’s test.

## Slide 5
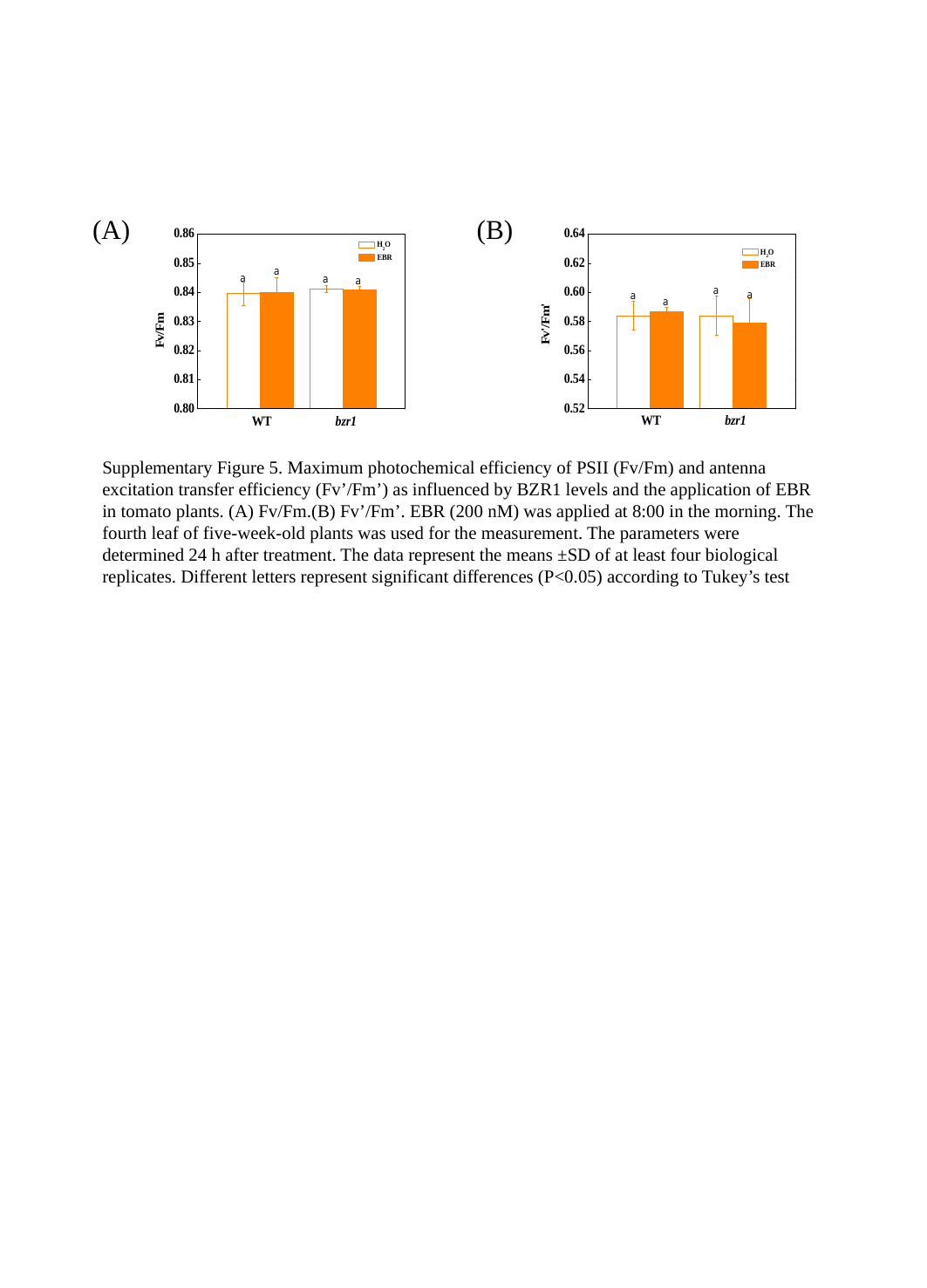

(A)
(B)
Supplementary Figure 5. Maximum photochemical efficiency of PSII (Fv/Fm) and antenna excitation transfer efficiency (Fv’/Fm’) as influenced by BZR1 levels and the application of EBR in tomato plants. (A) Fv/Fm.(B) Fv’/Fm’. EBR (200 nM) was applied at 8:00 in the morning. The fourth leaf of five-week-old plants was used for the measurement. The parameters were determined 24 h after treatment. The data represent the means ±SD of at least four biological replicates. Different letters represent significant differences (P<0.05) according to Tukey’s test

## Slide 6
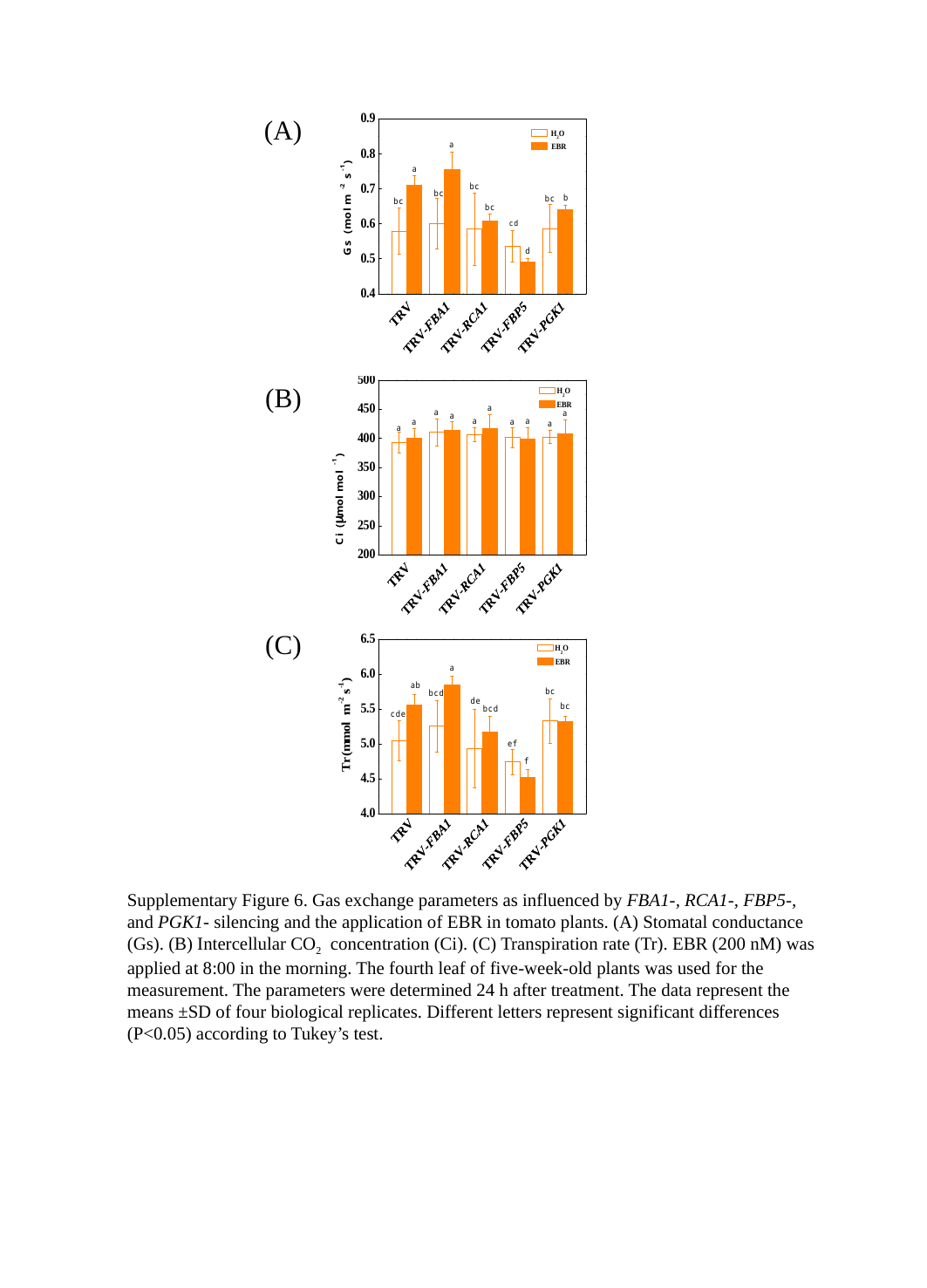

(A)
(B)
(C)
Supplementary Figure 6. Gas exchange parameters as influenced by FBA1-, RCA1-, FBP5-, and PGK1- silencing and the application of EBR in tomato plants. (A) Stomatal conductance (Gs). (B) Intercellular CO2 concentration (Ci). (C) Transpiration rate (Tr). EBR (200 nM) was applied at 8:00 in the morning. The fourth leaf of five-week-old plants was used for the measurement. The parameters were determined 24 h after treatment. The data represent the means ±SD of four biological replicates. Different letters represent significant differences (P<0.05) according to Tukey’s test.

## Slide 7
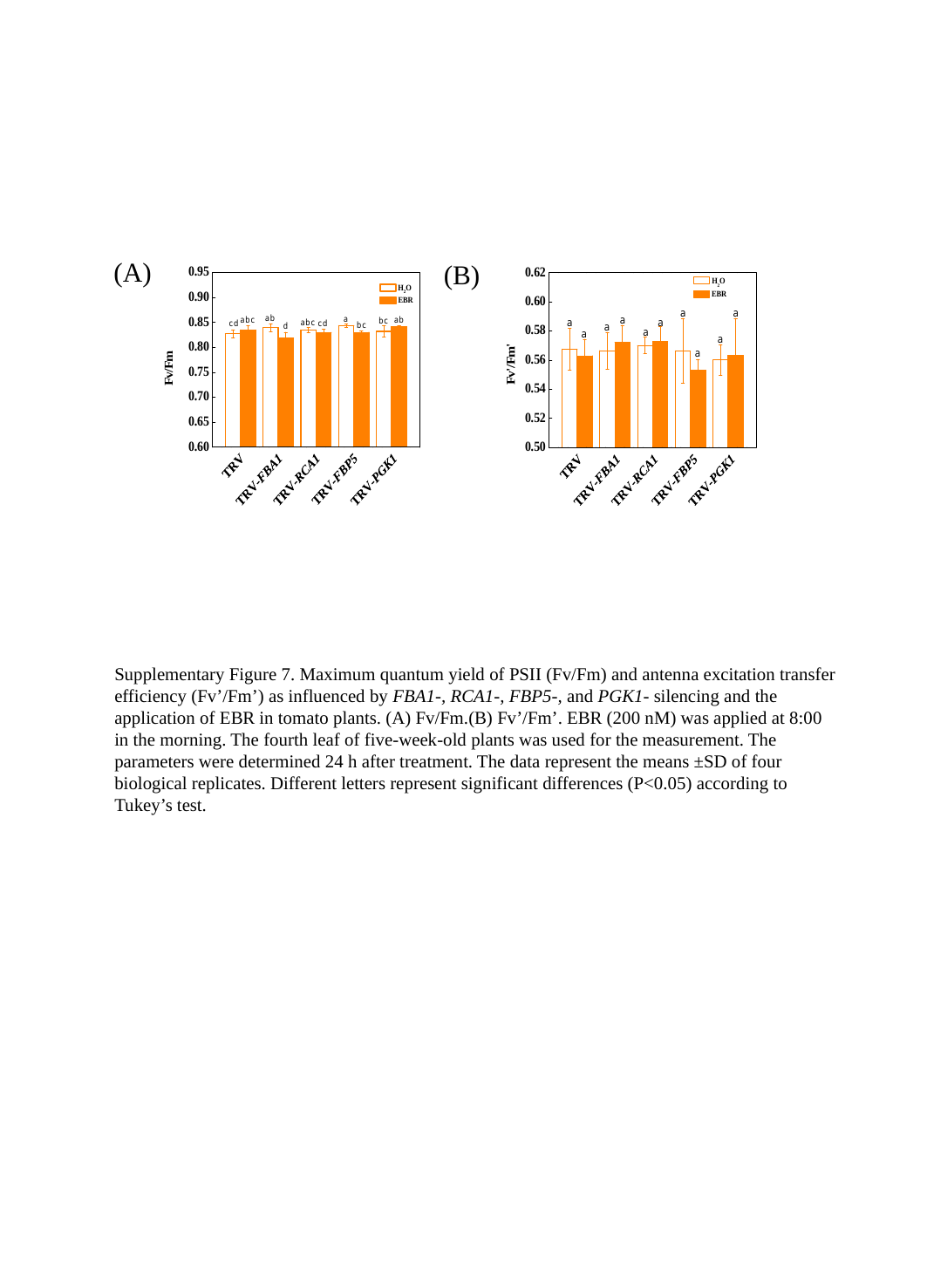

(A)
(B)
Supplementary Figure 7. Maximum quantum yield of PSII (Fv/Fm) and antenna excitation transfer efficiency (Fv’/Fm’) as influenced by FBA1-, RCA1-, FBP5-, and PGK1- silencing and the application of EBR in tomato plants. (A) Fv/Fm.(B) Fv’/Fm’. EBR (200 nM) was applied at 8:00 in the morning. The fourth leaf of five-week-old plants was used for the measurement. The parameters were determined 24 h after treatment. The data represent the means ±SD of four biological replicates. Different letters represent significant differences (P<0.05) according to Tukey’s test.

## Slide 8
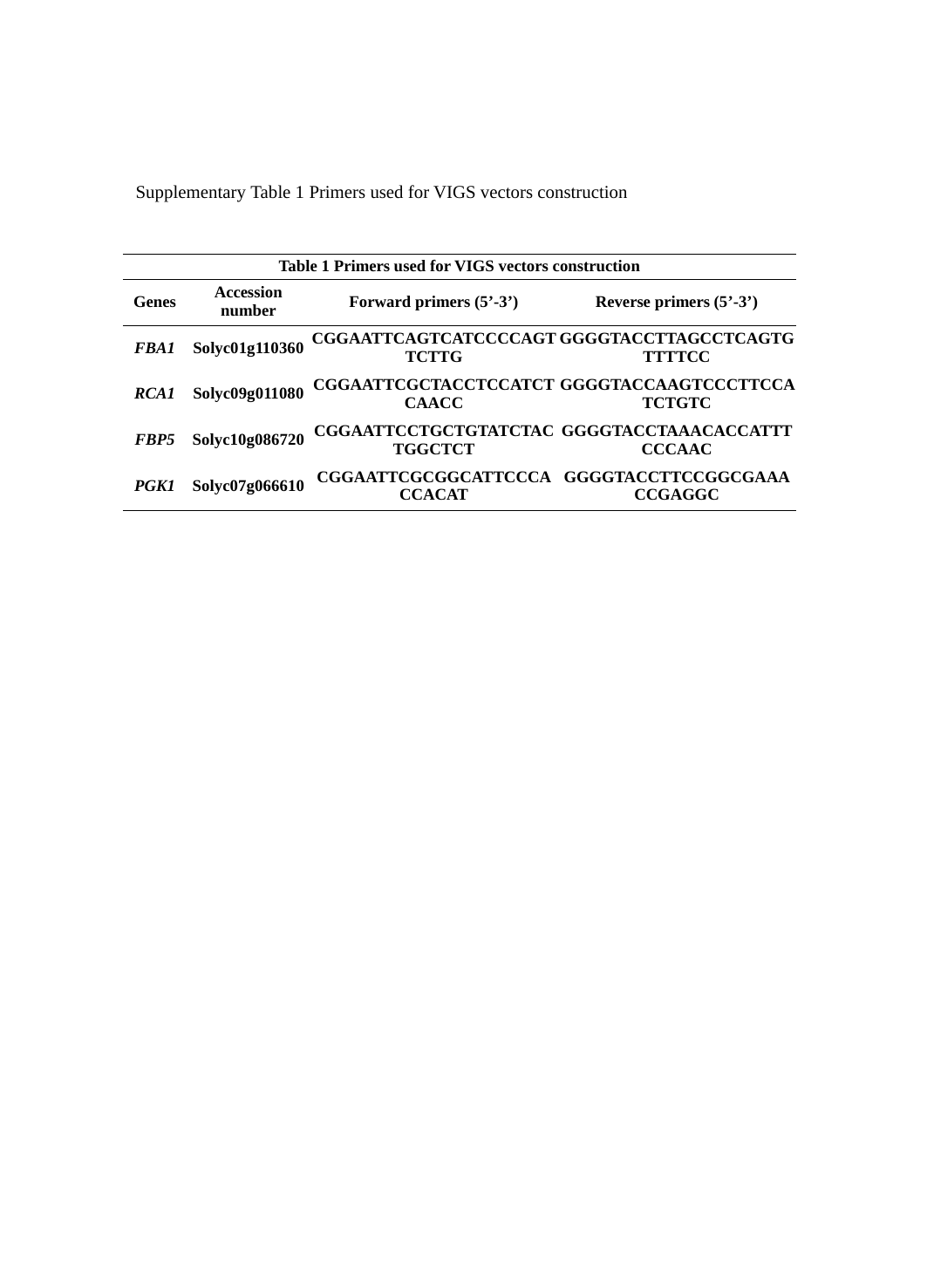

Supplementary Table 1 Primers used for VIGS vectors construction
| Table 1 Primers used for VIGS vectors construction | | | |
| --- | --- | --- | --- |
| Genes | Accession number | Forward primers (5’-3’) | Reverse primers (5’-3’) |
| FBA1 | Solyc01g110360 | CGGAATTCAGTCATCCCCAGTTCTTG | GGGGTACCTTAGCCTCAGTGTTTTCC |
| RCA1 | Solyc09g011080 | CGGAATTCGCTACCTCCATCTCAACC | GGGGTACCAAGTCCCTTCCATCTGTC |
| FBP5 | Solyc10g086720 | CGGAATTCCTGCTGTATCTACTGGCTCT | GGGGTACCTAAACACCATTTCCCAAC |
| PGK1 | Solyc07g066610 | CGGAATTCGCGGCATTCCCACCACAT | GGGGTACCTTCCGGCGAAACCGAGGC |

## Slide 9
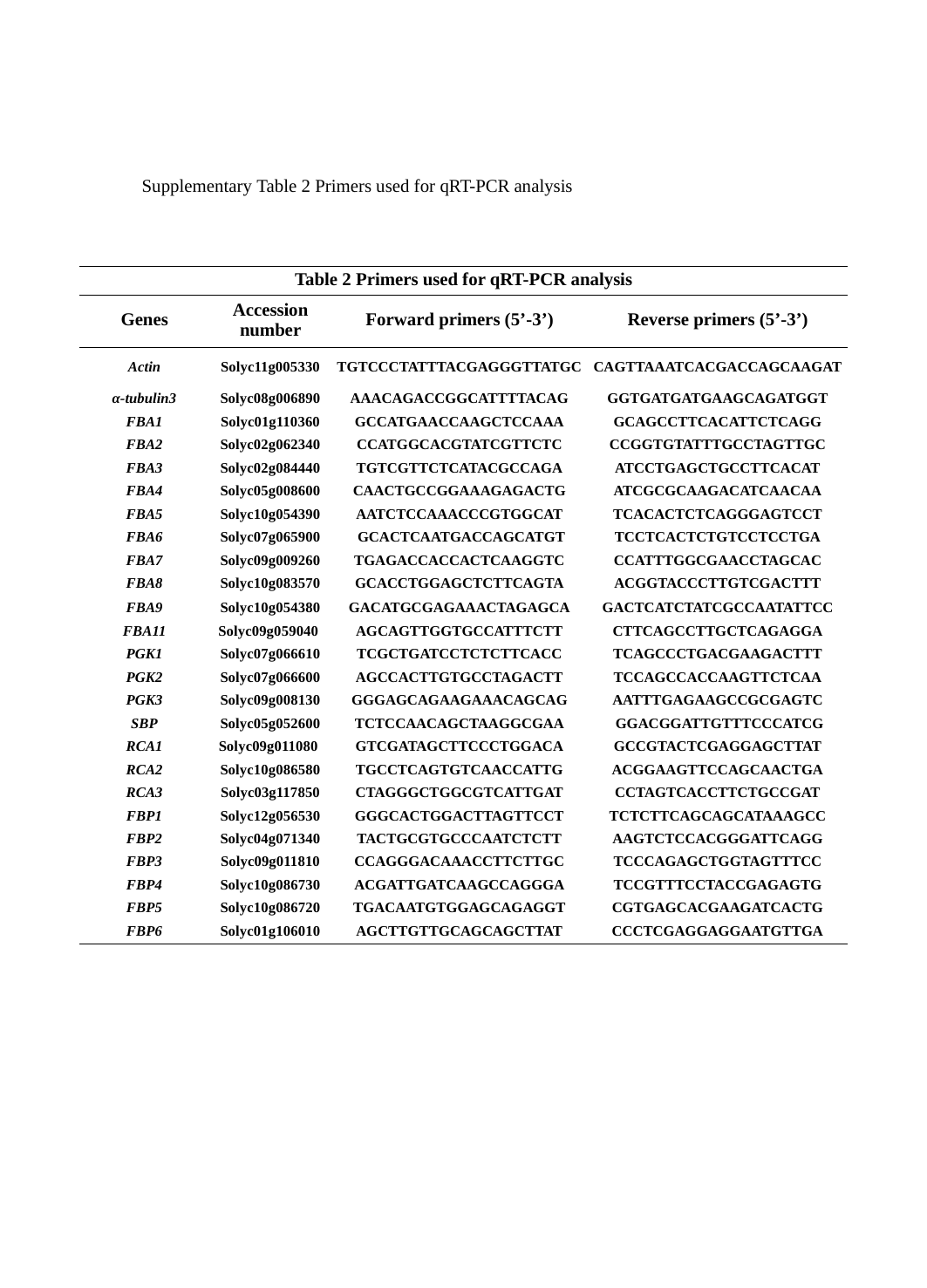

Supplementary Table 2 Primers used for qRT-PCR analysis
| Table 2 Primers used for qRT-PCR analysis | | | |
| --- | --- | --- | --- |
| Genes | Accession number | Forward primers (5’-3’) | Reverse primers (5’-3’) |
| Actin | Solyc11g005330 | TGTCCCTATTTACGAGGGTTATGC | CAGTTAAATCACGACCAGCAAGAT |
| α-tubulin3 | Solyc08g006890 | AAACAGACCGGCATTTTACAG | GGTGATGATGAAGCAGATGGT |
| FBA1 | Solyc01g110360 | GCCATGAACCAAGCTCCAAA | GCAGCCTTCACATTCTCAGG |
| FBA2 | Solyc02g062340 | CCATGGCACGTATCGTTCTC | CCGGTGTATTTGCCTAGTTGC |
| FBA3 | Solyc02g084440 | TGTCGTTCTCATACGCCAGA | ATCCTGAGCTGCCTTCACAT |
| FBA4 | Solyc05g008600 | CAACTGCCGGAAAGAGACTG | ATCGCGCAAGACATCAACAA |
| FBA5 | Solyc10g054390 | AATCTCCAAACCCGTGGCAT | TCACACTCTCAGGGAGTCCT |
| FBA6 | Solyc07g065900 | GCACTCAATGACCAGCATGT | TCCTCACTCTGTCCTCCTGA |
| FBA7 | Solyc09g009260 | TGAGACCACCACTCAAGGTC | CCATTTGGCGAACCTAGCAC |
| FBA8 | Solyc10g083570 | GCACCTGGAGCTCTTCAGTA | ACGGTACCCTTGTCGACTTT |
| FBA9 | Solyc10g054380 | GACATGCGAGAAACTAGAGCA | GACTCATCTATCGCCAATATTCC |
| FBA11 | Solyc09g059040 | AGCAGTTGGTGCCATTTCTT | CTTCAGCCTTGCTCAGAGGA |
| PGK1 | Solyc07g066610 | TCGCTGATCCTCTCTTCACC | TCAGCCCTGACGAAGACTTT |
| PGK2 | Solyc07g066600 | AGCCACTTGTGCCTAGACTT | TCCAGCCACCAAGTTCTCAA |
| PGK3 | Solyc09g008130 | GGGAGCAGAAGAAACAGCAG | AATTTGAGAAGCCGCGAGTC |
| SBP | Solyc05g052600 | TCTCCAACAGCTAAGGCGAA | GGACGGATTGTTTCCCATCG |
| RCA1 | Solyc09g011080 | GTCGATAGCTTCCCTGGACA | GCCGTACTCGAGGAGCTTAT |
| RCA2 | Solyc10g086580 | TGCCTCAGTGTCAACCATTG | ACGGAAGTTCCAGCAACTGA |
| RCA3 | Solyc03g117850 | CTAGGGCTGGCGTCATTGAT | CCTAGTCACCTTCTGCCGAT |
| FBP1 | Solyc12g056530 | GGGCACTGGACTTAGTTCCT | TCTCTTCAGCAGCATAAAGCC |
| FBP2 | Solyc04g071340 | TACTGCGTGCCCAATCTCTT | AAGTCTCCACGGGATTCAGG |
| FBP3 | Solyc09g011810 | CCAGGGACAAACCTTCTTGC | TCCCAGAGCTGGTAGTTTCC |
| FBP4 | Solyc10g086730 | ACGATTGATCAAGCCAGGGA | TCCGTTTCCTACCGAGAGTG |
| FBP5 | Solyc10g086720 | TGACAATGTGGAGCAGAGGT | CGTGAGCACGAAGATCACTG |
| FBP6 | Solyc01g106010 | AGCTTGTTGCAGCAGCTTAT | CCCTCGAGGAGGAATGTTGA |

## Slide 10
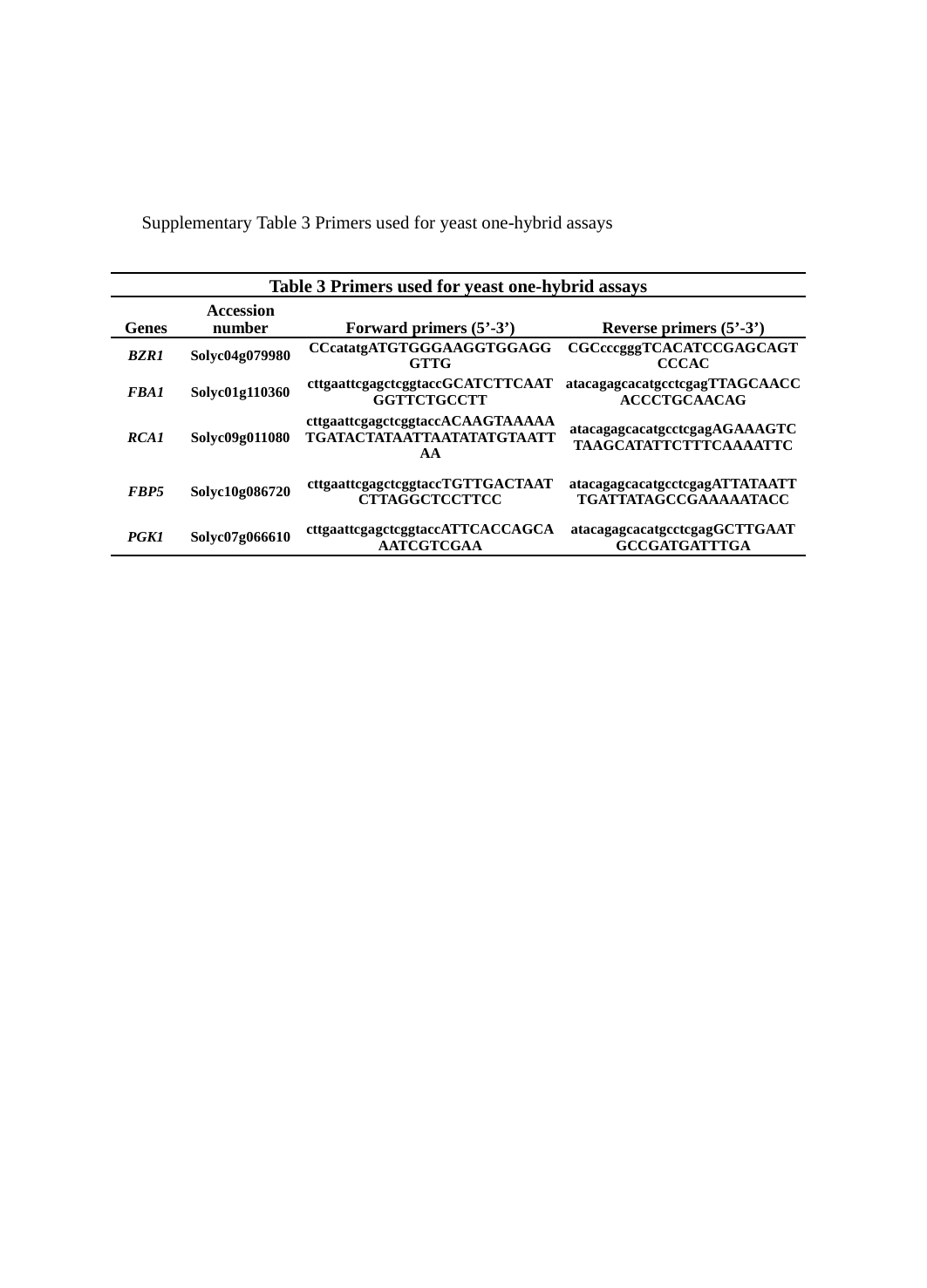

Supplementary Table 3 Primers used for yeast one-hybrid assays
| Table 3 Primers used for yeast one-hybrid assays | | | |
| --- | --- | --- | --- |
| Genes | Accession number | Forward primers (5’-3’) | Reverse primers (5’-3’) |
| BZR1 | Solyc04g079980 | CCcatatgATGTGGGAAGGTGGAGGGTTG | CGCcccgggTCACATCCGAGCAGTCCCAC |
| FBA1 | Solyc01g110360 | cttgaattcgagctcggtaccGCATCTTCAATGGTTCTGCCTT | atacagagcacatgcctcgagTTAGCAACCACCCTGCAACAG |
| RCA1 | Solyc09g011080 | cttgaattcgagctcggtaccACAAGTAAAAATGATACTATAATTAATATATGTAATTAA | atacagagcacatgcctcgagAGAAAGTCTAAGCATATTCTTTCAAAATTC |
| FBP5 | Solyc10g086720 | cttgaattcgagctcggtaccTGTTGACTAATCTTAGGCTCCTTCC | atacagagcacatgcctcgagATTATAATTTGATTATAGCCGAAAAATACC |
| PGK1 | Solyc07g066610 | cttgaattcgagctcggtaccATTCACCAGCAAATCGTCGAA | atacagagcacatgcctcgagGCTTGAATGCCGATGATTTGA |

## Slide 11
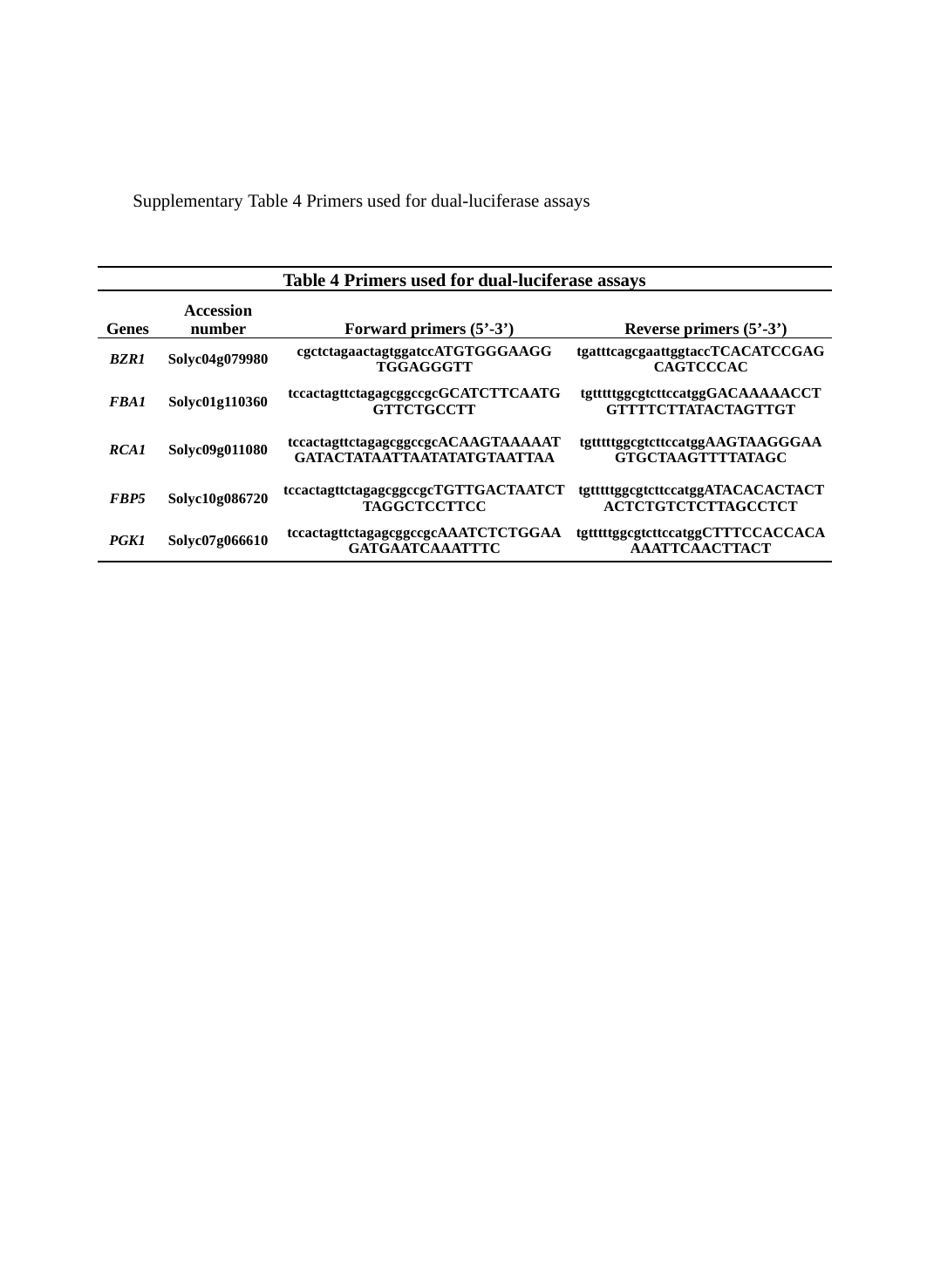

Supplementary Table 4 Primers used for dual-luciferase assays
| Table 4 Primers used for dual-luciferase assays | | | | |
| --- | --- | --- | --- | --- |
| Genes | Accession number | Forward primers (5’-3’) | | Reverse primers (5’-3’) |
| BZR1 | Solyc04g079980 | cgctctagaactagtggatccATGTGGGAAGG TGGAGGGTT | tgatttcagcgaattggtaccTCACATCCGAG CAGTCCCAC | |
| FBA1 | Solyc01g110360 | tccactagttctagagcggccgcGCATCTTCAATGGTTCTGCCTT | tgtttttggcgtcttccatggGACAAAAACCTGTTTTCTTATACTAGTTGT | |
| RCA1 | Solyc09g011080 | tccactagttctagagcggccgcACAAGTAAAAATGATACTATAATTAATATATGTAATTAA | tgtttttggcgtcttccatggAAGTAAGGGAAGTGCTAAGTTTTATAGC | |
| FBP5 | Solyc10g086720 | tccactagttctagagcggccgcTGTTGACTAATCTTAGGCTCCTTCC | tgtttttggcgtcttccatggATACACACTACTACTCTGTCTCTTAGCCTCT | |
| PGK1 | Solyc07g066610 | tccactagttctagagcggccgcAAATCTCTGGAAGATGAATCAAATTTC | tgtttttggcgtcttccatggCTTTCCACCACAAAATTCAACTTACT | |

## Slide 12
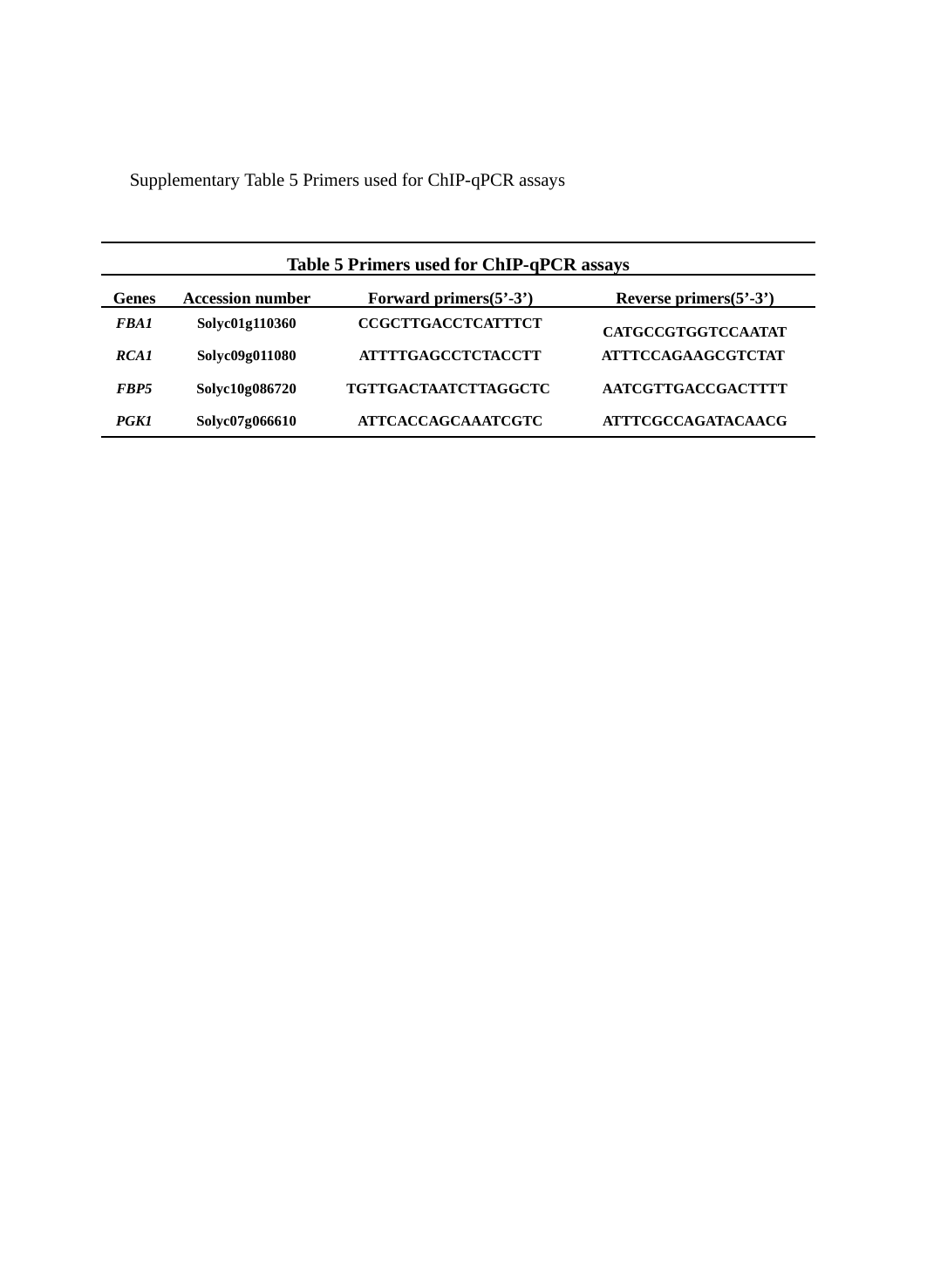

Supplementary Table 5 Primers used for ChIP-qPCR assays
| Table 5 Primers used for ChIP-qPCR assays | | | |
| --- | --- | --- | --- |
| Genes | Accession number | Forward primers(5’-3’) | Reverse primers(5’-3’) |
| FBA1 | Solyc01g110360 | CCGCTTGACCTCATTTCT | CATGCCGTGGTCCAATAT |
| RCA1 | Solyc09g011080 | ATTTTGAGCCTCTACCTT | ATTTCCAGAAGCGTCTAT |
| FBP5 | Solyc10g086720 | TGTTGACTAATCTTAGGCTC | AATCGTTGACCGACTTTT |
| PGK1 | Solyc07g066610 | ATTCACCAGCAAATCGTC | ATTTCGCCAGATACAACG |

## Slide 13
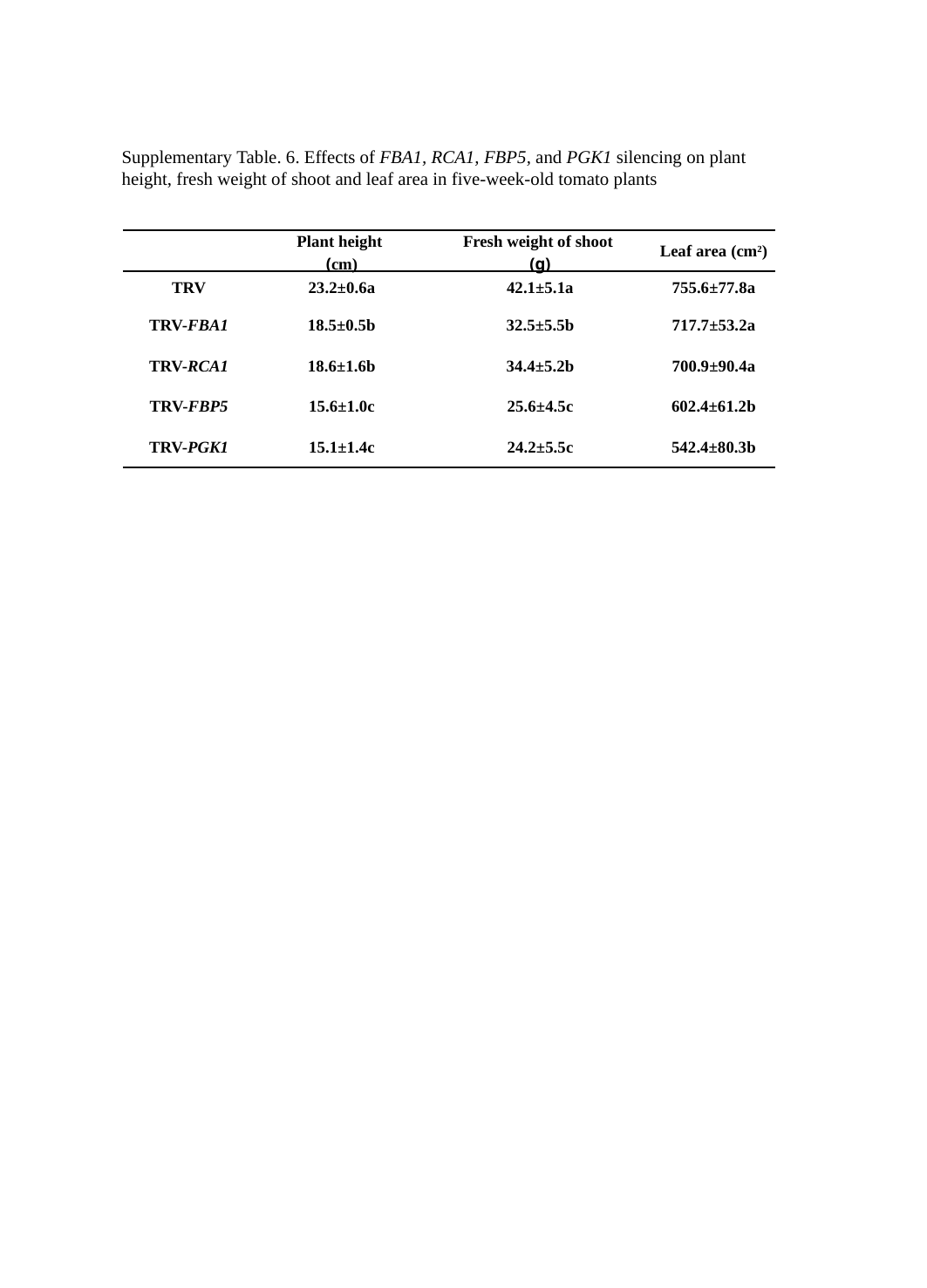

Supplementary Table. 6. Effects of FBA1, RCA1, FBP5, and PGK1 silencing on plant height, fresh weight of shoot and leaf area in five-week-old tomato plants
| | Plant height (cm) | Fresh weight of shoot (g) | Leaf area (cm2) |
| --- | --- | --- | --- |
| TRV | 23.2±0.6a | 42.1±5.1a | 755.6±77.8a |
| TRV-FBA1 | 18.5±0.5b | 32.5±5.5b | 717.7±53.2a |
| TRV-RCA1 | 18.6±1.6b | 34.4±5.2b | 700.9±90.4a |
| TRV-FBP5 | 15.6±1.0c | 25.6±4.5c | 602.4±61.2b |
| TRV-PGK1 | 15.1±1.4c | 24.2±5.5c | 542.4±80.3b |
